# Oligolysine-based coating protects DNA nanostructures from low-salt denaturation and nuclease degradation

**DOI:** 10.1038/ncomms15654

**Published:** 2017-05-31

**Authors:** Nandhini Ponnuswamy, Maartje M. C. Bastings, Bhavik Nathwani, Ju Hee Ryu, Leo Y. T. Chou, Mathias Vinther, Weiwei Aileen Li, Frances M. Anastassacos, David J. Mooney, William M. Shih

**Affiliations:** 1 Department of Cancer Biology, Dana-Farber Cancer Institute, 450 Brookline Avenue, Boston, Massachusetts 02215, USA; 2 Department of Biological Chemistry and Molecular Pharmacology, Harvard Medical School, Boston, Massachusetts 02115, USA; 3 Wyss Institute for Biologically Inspired Engineering at Harvard, Boston, Massachusetts 02115, USA; 4 Center for Theragnosis, Biomedical Research Institute, Korea Institute of Science and Technology, Seoul 02792, Republic of Korea; 5 Centre for DNA Nanotechnology, Interdisciplinary Nanoscience Center, iNANO, Aarhus University, Gustav Wieds Vej 14, 8000 Aarhus C, Denmark; 6 School of Engineering and Applied Sciences, Harvard University, Cambridge Massachusetts 02138, USA

## Abstract

DNA nanostructures have evoked great interest as potential therapeutics and diagnostics due to ease and robustness of programming their shapes, site-specific functionalizations and responsive behaviours. However, their utility in biological fluids can be compromised through denaturation induced by physiological salt concentrations and degradation mediated by nucleases. Here we demonstrate that DNA nanostructures coated by oligolysines to 0.5:1 N:P (ratio of nitrogen in lysine to phosphorus in DNA), are stable in low salt and up to tenfold more resistant to DNase I digestion than when uncoated. Higher N:P ratios can lead to aggregation, but this can be circumvented by coating instead with an oligolysine-PEG copolymer, enabling up to a 1,000-fold protection against digestion by serum nucleases. Oligolysine-PEG-stabilized DNA nanostructures survive uptake into endosomal compartments and, in a mouse model, exhibit a modest increase in pharmacokinetic bioavailability. Thus, oligolysine-PEG is a one-step, structure-independent approach that provides low-cost and effective protection of DNA nanostructures for *in vivo* applications.

## Abstract (web summary)

The instability of DNA nanostructures in physiological environments has hampered their use as therapeutics and diagnostic agents in *in vivo* applications. Here, the authors show that coating DNA origami with oligolysine-PEG moieties improves their pharmacokinetic properties in mouse models.

DNA nanostructures (DNs) can be programmed to fold into prescribed spatial configurations^1^,^2^,^3^,^4^,^5^,^6^, functionalized site specifically with a wide variety of guests^7^ and engineered to undergo allosteric conformational changes^8^,^9^,^10^,^11^. For application to diagnostics and therapeutics, DNs can impart these useful properties, such as controlled shape, addressability and responsiveness, to other nanomaterials—for example, liposomes, polymeric or metallic particles, proteins—and thereby augment their functionality. Furthermore, DNs are biodegradable and biocompatible.

However, current biomedical applications of DNs are hindered by two main challenges. First, stabilization of most DNs requires ∼5–20 mM divalent cations (for example, Mg^2+^) to overcome electrostatic repulsion between closely packed DNA phosphate anions. Therefore, most DNs exhibit poor structural integrity in physiological fluids, which typically contain low levels of Mg^2+^ and Ca^2+^ (for example, ∼0.4 mM each in RPMI-1640)^12^. Second, nuclease activity results in rapid degradation during incubation of these structures in freshly prepared cell medium containing 10% fetal bovine serum (FBS) at 37 °C^13^,^14^,^15^,^16^. Intravenous injection of a fluorescently labelled DN results in rapid clearance similar to that of a control oligonucleotide^17^. Thus, DNs must first be stabilized against Mg^2+^ depletion and nuclease degradation before they can be effective for most biomedical applications.

Several approaches have been investigated to address these challenges. Cassinelli *et al*.^17^ have demonstrated the use of click chemistry to catenate constituent tiles of six helix tubes, which renders these structures stable at low ionic strength; Benson *et al*.^18^ and Veneziano *et al*.^19^ have reported wireframe architectures for DNA origami that survive at physiological divalent cation concentrations. Coating of DNs with cowpea chlorotic mottle virus capsid proteins^20^ or with cationic poly(2-dimethylamino-ethylmethacrylate) (PDMAEMA)-based polymers^21^ has been reported; however, this coating appeared to induce aggregation of DNs as monitored by negative-stain transmission electron microscopy (TEM). Our group has previously reported virus-inspired encapsulation of DN in liposomes, which protects the structure from nuclease degradation and enhances *in vivo* circulation time^22^. However, this method requires attaching multiple lipid–DNA conjugates on DNs at defined positions and extensive protocol optimization to template bilayer self-assembly without aggregation or multimerizaton, which makes the process time consuming. Although successful in addressing individual challenges, each of these methods hence remain labour/cost intensive or are structure dependent. A need therefore remains for an easy, scalable and structure-independent method to stabilize DNs against commonly encountered *in vivo* threats.

We report here that coating DNs with oligolysine provides stability against denaturation at physiological Mg^2+^ concentrations without noticeable distortion or aggregation of the structure (as monitored by negative-stain TEM). Coating is driven by electrostatic interactions and is achieved simply by mixing stock solutions of DNs and oligolysine at appropriate stoichiometric ratios followed by incubation at room temperature. However, coating with oligolysine only modestly protects DNs against nucleases. Refining our approach, we show that coating with oligolysine conjugated to polyethylene glycol (PEG) significantly slows down nuclease degradation. Furthermore, we use a fluorescence resonance energy transfer (FRET)-based assay to demonstrate structural integrity of the coated nanostructures within cellular compartments.

## Results

### Stabilization of DNs against low-salt denaturation

**Figure 1:**
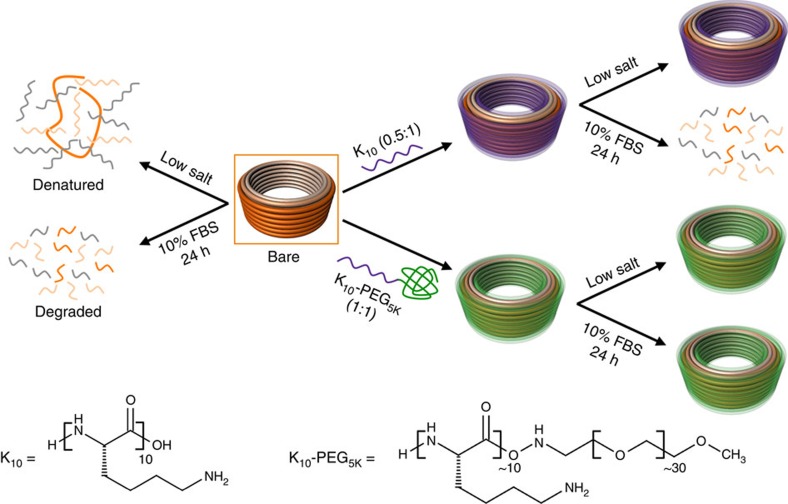
Protection of DNs from low salt and nucleases. The schematic represents the fate of bare and coated DNs in physiological buffers at 37 °C containing low salt and/or 10% FBS. Bare DN rapidly denatures at low salt and degrades in freshly prepared cell medium containing 10% FBS. Low-salt-induced denaturation and nuclease degradation can be overcome by coating the DNs with positively charged peptides such as K_10_ or K_10_–PEG_5K_ at an N:P of 0.5:1 and 1:1, respectively. The oligolysine peptide has α-lysine side chains.

We hypothesized that electrostatic repulsions in DNs due to Mg^2+^ depletion could be stabilized by using polyamines as a substitute for divalent cations (schematic with oligolysines as stabilizing agent shown in Fig. 1), as they would remain bound after dilution into physiological buffers (for example, see Supplementary Table 1 for RPMI-1640 composition), due to their much stronger binding. However, polyamines are known to condense DNA into compact nanoparticles; therefore, one concern is that polyamine binding could crush or deform DNs and thereby compromise their intricate three-dimensional shapes important for their functionality. A second concern is that polyamine binding could lead to aggregation of DNs.

**Figure 2:**
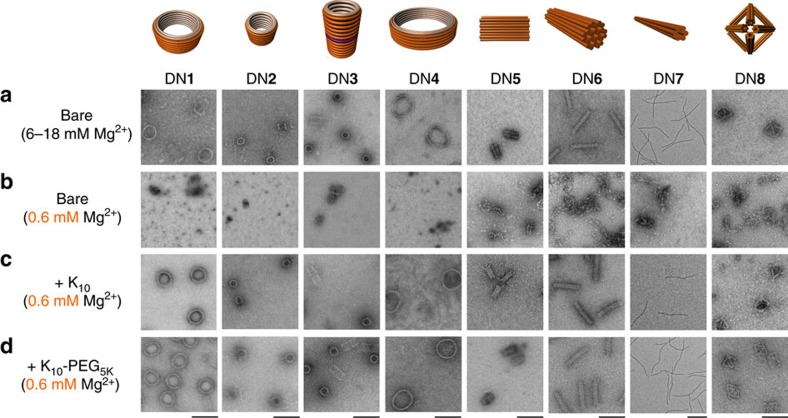
Low-magnesium stability of bare and coated DNs. (**a**) Negative-stain TEM images of bare DNs DN**1**–DN**8** in folding buffer (5 mM Tris, 1 mM EDTA, 6–18 mM Mg^2+^). Negative-stain TEM images of (**b**) bare DN**1**–DN**8**, (**c**) K_10_-coated DN**1**–DN**8**, (**d**) K_10_–PEG5_K_-coated DN**1**–DN**8** diluted in physiological buffer (RPMI-1640) such that the final Mg^2+^ concentration is ∼0.6 mM. All scale bars are 100 nm, except for DN7 (200 nm).

We first evaluated the structural integrity at low Mg^2+^ of a model DN that has the shape of a barrel (DN**1** in Fig. 2) using a set of various polyamines: spermidine, spermine, oligoarginines of length 6, oligolysines of length 6 and 10 kDa branched polyethyleneimine (PEI). All the polyamines are positively charged at physiological pH and have the ability to interact with DNA electrostatically (Supplementary Fig. 1). Coating of DN**1** was achieved by mixing DN**1** with a solution of the polyamine at the desired N:P ratio (nitrogen in amines to phosphorus in DNA) and incubating at room temperature for 30 min. To assess the structural integrity of DN**1** in physiological buffers, bare and polyamine-coated DN**1** were diluted in standard RPMI medium (133 mM Na^+^, 5.3 mM K^+^, 0.4 mM Mg^2+^ and 0.4 mM Ca^2+^) such that the final Mg^2+^ concentration was ∼0.6 mM. The solution was then heated at 37 °C for 1 h in a thermal cycler and then analysed using agarose gel electrophoresis (AGE) and TEM.

Analysis of the bare DN**1** at physiological levels of Mg^2+^ ions revealed that its structure was destroyed beyond recognition (assessed via TEM, Fig. 2) and with dissociation of staple strands from the scaffold strand (assessed via AGE, Supplementary Fig. 2). Structural integrity at low salt could be preserved by prior incubation with spermine and spermidine, but only at N:P ratios of 50:1 and 100:1, respectively. However, these samples denatured after overnight dialysis at room temperature into zero-Mg^2+^ buffer (5 mM Tris, 1 mM EDTA pH 8.0), presumably due to dissociation of the polyamines into the free solution. This result encouraged us to try longer polyamines to obtain stronger binding and therefore longer residence times, as well as better control over bound N:P ratios. Pre-incubation of DN**1** with PEI led to destruction of structural integrity due to compaction as observed by TEM, likely to be caused by strong multivalent binding (Supplementary Figs 2 and 3). In contrast, pre-incubation with oligoarginines protected against low-salt denaturation, however, at the cost of significant structural deformation (that is, barrel projection takes the appearance of a polygon instead of a circle (Supplementary Fig. 3).

We obtained the best results by far with oligolysine-coated DN**1**, which withstood divalent cation depletion at 37 °C and maintained structural integrity as observed via TEM. Different lengths of oligolysines K_*n*_ (where *n* is the number of L-lysine monomers; *n*=4, 6, 8, 10, 12, 16 and 20) were investigated (Supplementary Figs 4–6). We observed that shorter oligolysines (*n*<10) required high N:P ratio for stabilization of DNs, owing to their weaker binding. Longer oligolysines (*n*>10), on the other hand, were required in only sub-stoichiometric amounts for stabilization due to enhanced binding, however, at the cost of increased aggregation of DNs. The best results were observed for K_10_, where the preservation of structure could be achieved at an N:P ratio of 0.5:1. Some aggregates were observed by TEM and AGE, but most of these separated after the sample was diluted to 1 nM in RPMI-1640 and incubated at 37 °C for 1 h.

The stability induced by K_10_ at low divalent-cation concentration was further tested with other DN designs (labelled DN**2**–DN**8**; Fig. 2). These nanostructures were chosen as a representative set to highlight the generality of the approach against variations in structural features such as curvature, the presence of a cavity, charge density, crossover density and size (AGE and TEM analysis in Supplementary Figs 7 and 8). The unstabilized DNs required 6–18 mM Mg^2+^ to maintain their intact structures. In all the cases, upon dilution in standard RPMI medium, the bare structure denatured to scaffold and staple strands, as observed via AGE and TEM. In contrast, DNs coated with K_10_ were able to maintain complete structural integrity even after overnight dialysis into zero-Mg^2+^ buffer (5 mM Tris, 1 mM EDTA pH 8.0; Supplementary Figs 9–13).

### Stability of DNs against nuclease degradation

Encouraged by our results towards Mg^2+^ depletion, we moved on to tackle the second main challenge, which was to prevent rapid nuclease degradation of DNs in serum. Previous studies have reported that positively charged polyamines can condense DNA into nanoparticles that are protected from digestion by nucleases, a behaviour that has been exploited extensively in gene-delivery applications^23^. Therefore, we next investigated whether K_10_ coating protects DNs from nuclease digestion, despite not crushing them into condensed particles. For this and subsequent experiments, we focused on DN**1** as a model system; we selected DN**1** as a challenging test case for nuclease resistance, as it has very high surface area to mass ratio.

**Figure 3:**
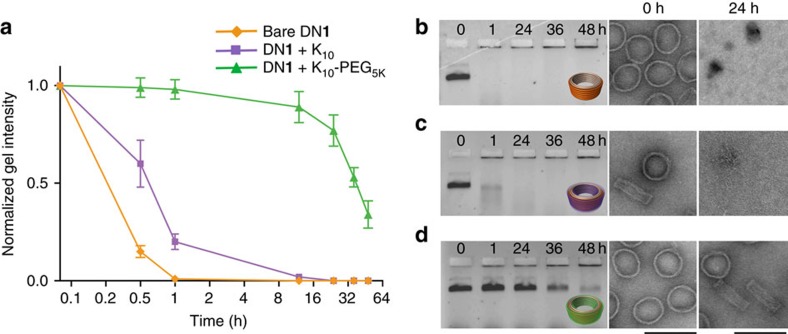
Time course of nuclease degradation of bare and coated DN**1**. Digestion was assayed in freshly prepared cell medium (RPMI-1640 containing 10% FBS) at 37 °C. (**a**) Normalized agarose gel intensity vs. time for bare, K_10_ coated and K_10_–PEG_5K_-coated DN**1**. Time (hours) is plotted with log_2_ scaling and error bars represent s.d. (*n*=3 s). Agarose gel and negative-stain TEM images at 0 h and 24 h for (**b**) bare DN**1**, (**c**) K_10_-coated DN**1** and (**d**) K_10_–PEG_5K_-coated DN**1** incubated in freshly prepared primary BMDC medium at 37 °C. Scale bars, 100 nm.

To assess the kinetics of nuclease digestion, the bare and K_10_ (N:P=0.5:1)-coated DN**1** were incubated in RPMI supplemented with 10% FBS at 37 °C for different time points. After incubation, the samples were analysed by AGE and TEM, and compared with a control incubated in RPMI+10 mM Mg^2+^, but lacking FBS. Bare DN**1** reached 50% degradation by ∼5 min in standard RPMI buffer, whereas it did not reach 50% degradation until ∼55 min in RPMI supplemented with 10 mM Mg^2+^. Likewise, K_10_-coated DN**1** (N:P=0.5:1) did not reach 50% degradation until ∼50 min. Therefore, the K_10_ treatment protects DN**1** to the same degree (∼5–10-fold enhanced resistance to nuclease degradation) as Mg^2+^ (Fig. 3). We hypothesize this is primarily due to the preservation of overall structure, and that low-salt denatured structures are better substrates for serum nucleases, perhaps due to their more open and accessible configurations (Supplementary Figs 14–17).

Seeking further improved resistance against nuclease digestion, we explored the use of a K_10_–PEG_*n*K_ co-polymer where the amino terminus of K_10_ was covalently functionalized with either a 1, 5 or 20 K PEG_*n*_ (*n*=1, 5 and 20 K). PEGylation of K_10_ was chosen because previous studies have reported an improvement in stability and increase *in vivo* circulation times post PEGylation of nanoparticles^24^. Furthermore, PEGylation might discourage aggregation of particles and therefore enable the use of higher N:P ratios that otherwise would lead to such aggregation yet might provide greater nuclease resistance. Coating of DN**1** with K_10_–PEG_*n*K_ was performed using the same protocol as described above for K_10_, but with a N:P ratio of 1:1.

K_10_–PEG_*n*K_-coated DN**1** was imaged via TEM, to ensure that the dense coating of K_10_–PEG_*n*K_ did not compromise structural integrity in low-salt physiological buffers. TEM images showed that DN**1** coated with K_10_–PEG_5K or 20K_ at a N:P ratio of 1:1 were intact and displayed no visible difference when compared with the bare nanostructures (Supplementary Fig. 18). In contrast, DN**1** coated with K_10_–PEG_1K_ at a N:P ratio of 1:1 were extensively aggregated (Supplementary Fig. 18). The K_10_–PEG_*n*K_-coated DNs are neutral at a N:P ratio of 1:1 and hence are retained in the well during AGE. Before AGE, any K_10_–PEG_*n*K_ coating can be removed if desired by incubating the sample with chondroitin sulfate, a negatively charged sulfated glycosaminoglycan, which electrostatically sequesters the positively charged K_10_–PEG_*n*K_ and allows the DNs to penetrate the gel matrix (Supplementary Fig. 9).

To assess whether these PEGylated oligolysine coatings would provide greater resistance against nuclease degradation, we incubated the K_10_–PEG_5K or 20K_-coated DN**1** (1 nM) in RPMI supplemented with 10% FBS at 37 °C for different time points. Before AGE, nucleases can be deactivated using 5 mM EGTA and 10% β-mercaptoethanol^25^ (Supplementary Fig. 19), and subsequently oligolysine shell can be removed by incubation with chondroitin sulfate to enable AGE analysis. Strikingly, AGE and TEM analysis showed that K_10_–PEG_5K_-coated DN**1** reached 50% degradation in ∼36 h compared with only 5 min for the bare DN**1**, corresponding to a ∼800 × enhancement in resistance to nuclease degradation (Fig. 3). K_10_–PEG_20K_-coated DN**1**, on the other hand, showed almost comparable nuclease protection (50% degradation at ∼24 h) compared with K_10_–PEG_5K_-coated DN**1** (Supplementary Fig. 20).

We also performed a DNase I titration assay, where bare and K_10_–PEG_5K_-coated DN**1** (1 nM) were incubated with increasing amounts of DNase I in standard RPMI medium for 1 h at 37 °C. Bare DN1 was ∼50% degraded in ∼0.5 U ml^−1^ of DNase I, as observed by AGE. In contrast, K_10_–PEG_5K-_coated DN**1** did not show significant degradation even in ∼500 U ml^−1^ of DNase I as observed by AGE and TEM, corresponding to a ∼1,000-fold increase in nuclease resistance (Supplementary Figs 21 and 22).

DNs such as DN**9** (mesh helix^19^; Supplementary Fig. 23), constructed using a polyhedral mesh architecture, are intact in low-salt buffers due to the low incidence of adjacent double helices and therefore exhibit greater nuclease resistance at low salt compared with multilayer DNs such as DN**1** (ref. 19). We also found that even DN**9** exhibit ∼fivefold greater nuclease resistance when coated with K_10_–PEG_5K_ (Supplementary Fig. 24). This result suggests that the nuclease resistance offered by K_10_–PEG_5K_ is not only due to the preservation of structural integrity of DNs in low-salt buffers, but also due in part to other factors such as steric shielding provided by the PEG layer that may reduce binding of nucleases to the DNA.

### Accessibility of surface receptors in K_10_–PEG_5K_-coated DNs

Next we addressed whether K_10_–PEG_5K_ coating of DNs blocks addressable surface features from functionalization (the attachment of 5K PEG at a N:P of 1:1 increases the thickness of the nanostructure by 2.4±1.3 nm as measured by dynamic light scattering); as a proxy, we assayed the ability of single-stranded DNA (ssDNA) handles protruding of the surface of DNs to interact with ssDNA anti-handles in solution. For this purpose, we incorporated 12 inward-facing 21mer ssDNA handles on the inside of DN**1**, to be bound by Alexa-750-labelled DNA anti-handles recruited from bulk solution (Supplementary Figs 25 and 26). We incubated DN**1** containing the ssDNA handles, bare versus K_10_-coated versus K_10_–PEG_5K_-coated, with Alexa-750-labelled DNA anti-handle for 2 h at 37 °C and monitored the incorporation of the Alexa 750 fluorophore into DN**1** using AGE. We observed that the incorporation efficiency of Alexa 750 was almost identical in all the three cases, implying that K_10_–PEG_5K_ coating of DNs allows accessibility to surface decorations.

To further demonstrate that targeting by receptors positioned on DNs is not inhibited by the K_10_–PEG_5K_ coating, we positioned 72 αvβ3-integrin-specific cyclic arginine-glycine-aspartate^26^ ligands strategically on the surface of DN**6**. We coated DN**6** with K_10_–PEG_5K_ and monitored cellular uptake into human umbilical vein endothelial cells by flow cytometry analysis. Our data shows that we are able to target surface integrin receptors on the cells and modestly enhance cellular uptake (Supplementary Fig. 27).

### Cellular uptake of K_10_–PEG_5K_-coated DN**1** into BMDCs

Thus far, we had demonstrated that coating of DNs with K_10_–PEG_5K_ at an N:P ratio of 1:1 stabilizes them against Mg^2+^ depletion and nuclease degradation. Towards cellular and downstream *in vivo* applications, we next investigated whether K_10_–PEG_5K_ coating is non-toxic and allows cellular uptake of DNs. For the subsequent experiments, we designated uncoated DN**1** as DN**1**_bare_ and K_10_–PEG_5K_-coated DN**1** as DN**1**_coated_.

We chose to observe the cellular uptake of DN**1**_bare_ and DN**1**_coated_ into mouse primary bone marrow-derived dendritic cells (BMDCs). BMDCs provide a challenging test case, as they have been reported to degrade endocytosed DNA with great efficiency^27^. We incorporated either 18 Cy5 or 18 Cy3 fluorophores into the inner DNA helices of DN**1**. The fluorophore-labelled DN**1** was incubated with BMDCs for 12 h and visualized using confocal fluorescence microscopy.

As expected, DN**1**_bare_ was not taken into the cells to a measurable extent; we infer that this is partly due to its rapid denaturation and degradation in the culture medium (Supplementary Fig. 28). In contrast, DN**1**_coated_ was internalized into dendritic cells (Supplementary Fig. 29) and, most importantly, we did not observe any measurable cytotoxicity (that is, signs of apoptosis) at maximum DN**1**_coated_ concentration of 20 nM (Supplementary Note 1 and Supplementary Figs 49 and 50). BMDCs incubated with DN**1**_coated_ exhibited punctate fluorescence indicative of DNs trapped in vesicles. Subsequently, we co-incubated BMDCs with Cy5-labelled DN**1**_coated_ and fluorescein-labelled transferrin, which is a marker for endosomal compartments. Similar punctate fluorescence was observed in both colour channels, suggesting that DNs were mostly resident within endosomal and endolysosomal compartments of cells (Supplementary Fig. 30).

### Structural integrity of DN inside cell compartments

The environment of cellular compartments can be drastically different from the outside medium and, therefore, it is of great interest to investigate the persistence and structural integrity of DNs within those compartments. To monitor the persistence of DNs within endosomal compartments of cells, we incubated Cy5-labelled DN**1**_coated_ (5 nM final DN**1** concentration, ∼90 nM Cy5 fluorophore) with BMDCs for 12 h and then washed the cells with culture medium to remove any excess DNs present in the medium. Subsequently, we monitored the decay in fluorescence intensity within cells for 24 h. We observed that fluorescence from DN**1**_coated_ displayed a half-life of ∼8 h post washing within cells. DNs either can be exported as intact structures or else first degraded and then exported; our experiments could not differentiate between these two export trajectories (Supplementary Fig. 31).

**Figure 4:**
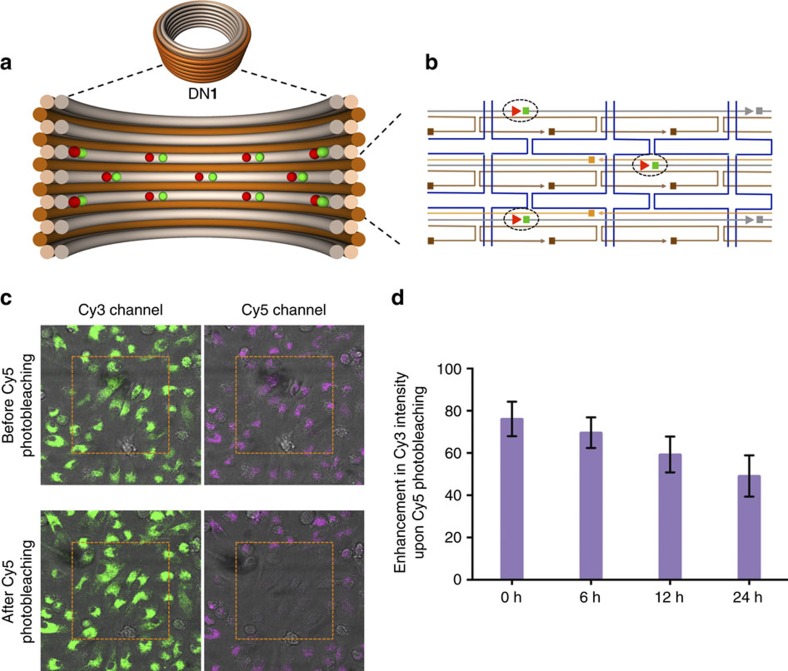
FRET assay of intracellular integrity of K_10_–PEG_5K_ coated DN1. (**a**) Schematic representation of the cross section of DN**1** functionalized with Cy3 (green) and Cy5 (red) fluorophores. (**b**) caDNAno^32^ diagram highlighting the position of fluorophores relative to the other staples (blue: p7308 scaffold; brown: outer staples; orange: inner-middle staples; grey: inner staples) (**c**) Confocal images of primary BMDCs from mice incubated with Cy3 and Cy5 fluorophore labelled DN**1**. (**d**) Enhancement in Cy3 intensity upon Cy5 photobleaching of BMDCs measured using confocal microscopy at 0, 6, 12 and 24 normalized to *in vitro* measurements. Error bars represent s.d. (*n*=2).

To further assess the structural integrity of DNs within cells, we designed a FRET assay based on donor-fluorescence enhancement induced by acceptor photobleaching^28^; this is a commonly used technique to enhance the sensitivity of FRET detection. For this purpose, every alternate DNA oligonucleotide binding to the inner DNA helices (Fig. 4) of DN**1**_coated_ was either functionalized at its 3′-end with an acceptor Cy5 fluorophore or at its 5′-end with a donor Cy3 fluorophore. The design is such that in an intact structure, the distance between adjacent Cy3 and Cy5 fluorophores is well under the estimated Förster distance of ∼5.6 nm; the distance between non-adjacent fluorophores is ∼8 nm. Energy transfer from a Cy3 donor to its adjacent Cy5 acceptor will result in the quenching of Cy3 fluorescence. After all Cy5 acceptors are photobleached using strong laser intensity, energy transfer from Cy3 to Cy5 is destroyed, resulting in the effective enhancement of the Cy3 donor signal. Alternatively, for structures where the close arrangement between Cy3 to Cy5 had previously been disrupted due to prior denaturation and/or degradation, then photobleaching of Cy5 will not result in any further Cy3 fluorescence enhancement. In cells, the amount of Cy3 fluorescence enhancement due to photobleaching therefore can be used to infer the amount of intact structures within the cells.

To validate that our assay could be used to monitor structural integrity, first we verified the experiment on a single-molecule level in a cell-free environment by total internal reflectance fluorescence (TIRF) microscopy. For these measurements, Cy3/Cy5-labelled DN**1**_coated_ was functionalized with 12 biotins and deposited on a streptavidin-coated glass surface and incubated in BMDC culture medium. After ∼99% laser photobleaching of Cy5 fluorophore, a 102.5%±2.4 enhancement in Cy3 fluorescence was observed (Supplementary Fig. 32). As a negative control, when Cy3 and Cy5 fluorophores in DN**1**_coated_ were placed at distances>Förster distance, no appreciable enhancement in Cy3 fluorescence was observed (Supplementary Fig. 33). Furthermore, we verified in bulk studies that Cy3 fluorescence enhancement could be abolished by pre-incubation with DNase I (which degrades the structure) or PEI (which crushes the structure) (Supplementary Note 2 and Supplementary Figs 51–53). Interestingly, we found that even after 50% of the Cy3 fluorescence enhancement had been pre-degraded by DNase I, structural integrity (assessed by AGE and TEM) was retained. This is consistent with a model where DNs can sustain a large number of local strand scissions before catastrophic loss of structure occurs.

Encouraged by our cell-free results, we incubated Cy3/Cy5-labelled DN**1**_coated_ with BMDCs for 12 h and washed the cells to remove excess structures. We then monitored the enhancement of Cy3 fluorescence upon Cy5 photobleaching at 0, 6, 12 and 24 h time points (Supplementary Figs 34–37). In this experiment, we observed enhancement of cellular donor fluorescence of 76.13%±8.2 at 0 h, 69.63%±7.3 at 6 h, 59.27%±8.5 at 12 h and 49.14%±9.8 at 24 h, respectively (Fig. 4).

The 20% decrease in the enhancement of donor fluorescence at 0 h post washing relative to the cell-free measurement is indicative of either our inability to accurately replicate endosomal and endolysosomal environments, or that some of the DN**1**_coated_ are already partially degraded, or both. At 24 h post washing, 49.14%±9.8 enhancement of donor fluorescence was observed, suggesting that structurally intact DN**1**_coated_ are still present within cells. We note here again that our cell-free experiments (Supplementary Note 2 and Supplementary Fig. 51) demonstrated that structures that display 50% of maximum fluorescence enhancement remained globally intact as indicated by agarose-gel and TEM analysis; this implies that most particles retained their structural integrity in endosomes at our 24 h post-washing time point. A model consistent with these observations is that most particles that have globally collapsed are rapidly removed from the endosomal compartments; as discussed above, ∼87% of uptaken DNs have been cleared from endosomes by the 24 h post-wash time point.

### Biodistribution of DN1_coated_ in mice

**Figure 5:**
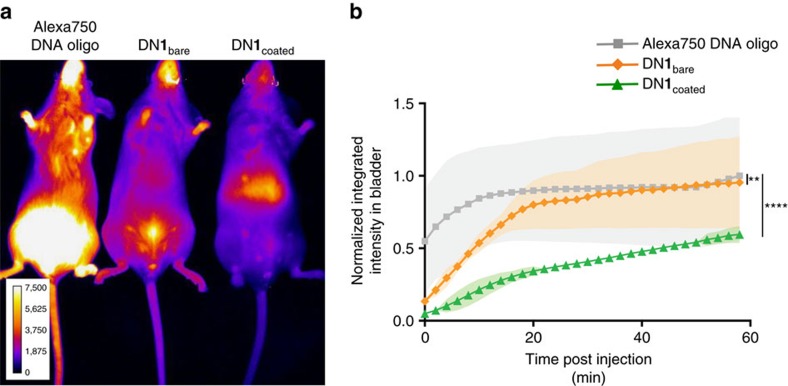
*In vivo* optical imaging for biodistribution and pharmacokinetics of DN1_coated_. (**a**) Fluorescence images of mice injected with either Alexa750 DNA oligo or DN**1**_bare_ and DN**1**_coated_ functionalized with 36 Alexa750 fluorophores at 2 min post injection. (**b**) Mean fluorescence intensity in bladder versus time. (Shaded regions represent s.d., *n*=3; two-way analysis of variance test; **P<=0.01 and ****P<0.0001).

Finally, we profiled the biodistribution of Alexa 750-labelled DN**1**_coated_ injected retro-orbitally in mice and compared it with negative controls: Alexa 750-labelled DNA oligonucleotide and Alexa 750-labelled DN**1**_bare_. Alexa 750 fluorescence was monitored every 2 min for 1 h using Perkin Elmer *in vivo* imaging system (IVIS, Supplementary Fig. 38). Subsequently, organs were harvested and analysed individually (Supplementary Fig. 39). Both the DNA oligonucleotide and the DN**1**_bare_ displayed rapid renal clearance (as evidenced by accumulation in the bladder), with a half time of 5 min and 9 min, respectively (Fig. 5 and Supplementary Fig. 40). In contrast, DN**1**_coated_ was cleared more slowly with a half-life of ∼45 min; we hypothesize this improvement was due to the increased resistance of DN**1**_coated_ structures to denaturation and nuclease degradation, while in circulation.

## Discussion

Herein we have developed a method that overcomes two major challenges that limit the effective utilization of DNs *in vivo*. Coating of DNs with positively charged oligolysine such as K_10_ stabilizes them against denaturation at physiological divalent-ion concentrations and remarkably results in little or no distortion of the three-dimensional structure, as assessed by inspection of negative-stain TEM images. Coating with a PEGylated oligolysine co-polymer, K_10_–PEG_5K_ significantly stabilizes the DNs against nuclease degradation by ∼1,000-fold. K_10_–PEG_5K_-coated DNs are readily taken up into endosomes and endolysosomes of BMDC without any apparent cytotoxicity (that is, visible rounding of cells). A significant fraction of endocytosed DNs (∼13%) persist as intact structures within the cellular compartments even after 24 h. Preliminary mice experiments show modest improvement in circulation and biodistribution of DN**1**_coated_ relative to DN**1**_bare_. PEGylated oligolysine therefore offers a low-cost, highly effective method for protection of DNs for local therapeutic delivery and, potentially, for systemic therapeutic delivery applications, where modest circulation times are sufficient. In the future, longer circulation times may be achievable by additional surface decorations, for example, CD47 ‘don't eat me' signals^29^.

## Methods

### Materials

Tris/Borate/EDTA buffer, PCR tubes and 96-well PCR plates were purchased from VWR. Oligolysine (K_4_–K_10_ and R_6_) were purchased from Peptide 2.0 as crude in a 5 mg scale. Oligolysine K_10_–PEG_1K_, K_10_–PEG_5K_ and K_10_–PEG_20K_ were purchased from Alamada polymers in 100 mg scale. Agarose was purchased from Lonza. Magnesium chloride, sodium chloride, glycerol, Tris base, EDTA and Tween20 was purchased from Sigma-Aldrich. RPMI, PBS, FBS and penicillin–streptomycin were purchased from Gibco. Carbon Formvar grids and uranyl formate were purchased from Electron Microscopy Sciences. Amicon Ultra filtration devices and 3.5 K MWCO Slide-A-Lyzer mini dialysis devices were purchased from Fisher Scientific. DNA gel extraction spin column was purchased from Bio-Rad.

### Nanostructure synthesis and purification

The design-specific staple strands were purchased from IDT Technologies in 250 μM scale. The sequence of the staple strands and the design is reported in the Supplementary Information. The p7308 scaffold strand was produced from M13 phage replication in *Escherichia coli* and was endotoxin-purified using Triton X-114 as described previously^13^.

For the synthesis of DNs, 10 nM p7308 scaffold was mixed with tenfold excess of staples in TE buffer (5 mM Tris and 1 mM EDTA) containing 10–20 mM MgCl_2_. The amount of MgCl_2_ varied with the structure: DN**1** (10 mM), DN**2** (6 mM), DN**3** (10 mM), DN**4** (10 mM), DN**5** (10 mM), DN**6** (12 mM), DN**7** (14 mM), DN**8** (12 mM) and DN**9** (6 mM). The solutions were subjected to a thermal annealing ramp on a Tetrad 2 Peltier thermal cycler (Bio-Rad) according to the following schedule: incubate at 65 °C for 15 min, decrease to 50 °C, incubate at 50 °C for 6 h 30 min and decrease to 40 °C at 6 h 30 min °C^−1^. The quality of folding was analysed by AGE. Solutions of folded DN were concentrated tenfold using a 30k MWCO Amicon Ultra centrifugal filter device (Millipore) and then purified by glycerol gradient ultracentrifugation^30^. The 45% and 15% glycerol solutions were made in 1 × TE buffer containing the same levels of MgCl_2_ as required for folding. The glycerol fractions containing nanostructures was concentrated and buffer exchanged to remove glycerol using a 30k MWCO Amicon Ultra centrifugal filter device. Following purification, the stock solution was diluted appropriately for TEM imaging to verify quality. The stock concentration was determined by ultraviolet absorbance at 260 nm on a Nanodrop spectrophotometer (Thermo Scientific), assuming that *A*_260_=1 for 50 μg ml^−1^ DNs. Stock solutions were stored at 4 °C until use.

### Agarose gel electrophoresis

After folding, DNs were analysed by AGE (2% agarose, 0.5 × TBE 10 mM MgCl_2_, 1 × ethidium bromide) using Thermo Scientific EasyCast Mini Gel System apparatus. Ten microlitres of samples were mixed with 3 μl of 6 × loading buffer and were loaded into the agarose gel. The samples were allowed to migrate for 2–3 h (running buffer: 0.5 × TBE, 11 mM MgCl_2_; 65 V). The gel was imaged with Typhoon FLA 9000 (GE Healthcare Life Sciences). To recover nanostructures, the bands were visualized with ultraviolet light and cut out from the gel. Extracted bands were crushed and placed into a DNA gel extraction spin column (Bio-Rad). The nanostructure solution was recovered by centrifugation of the loaded column for 5 min at 13,000 *g*.

### Negative-stain TEM analysis

Three microlitres of DNs were pipetted onto a plasma-treated carbon Formvar grid (Electron Microscopy Sciences) and incubated for 1 min. The solution was wicked away onto filter paper and 7 μl of freshly prepared 2% uranyl formate (in H2O, w/v) was immediately added. This was incubated for 0.5 min and then wicked away by filter paper. Imaging was carried out on a JEOL 1400 TEM.

### Coating with K_10_ and K_10_–PEG_*n*K_

Ten microlitres of 10 nM DN**1**–DN**9** was mixed with 1 μl of oligolysine (K_10_ or K_10_–PEG_*n*K_) at a concentration where the desired N:P ratio (ratio of nitrogen in amines:phosphates in DNA) would be achieved and the sample incubated at room temperature for 30 min. K_10_–PEG_*n*K_ were purchased from Alamanda polymers, their polydispersity index from gel permeation chromatography is between 1.00 and 1.20. The number average molecular weight range by NMR as provided by the company for K_10_–PEG_1K_ is 2,200–3,100 Da, for K_10_–PEG_5K_ is 5,800–7,500 Da and for K_10_–PEG_20K_ is 18,000–22,000 Da.

### Magnesium depletion assays

Bare DNs and K_10_ or K_10_–PEG_5K_-coated DNs were diluted in RPMI-1640 medium (Gibco) such that the final Mg^2+^ concentration is 0.6 mM. The samples were incubated at 37 °C for 1 h on a Tetrad 2 Peltier thermal cycler (Bio-Rad) and analysed using AGE and TEM imaging. Alternatively, 100 μl bare DNs and K_10_ or K_10_–PEG_5K_-coated DNs were added to 3.5 K MWCO Slide-A-Lyzer mini dialysis units (Fisher Scientific) and dialysed overnight at room temperature into zero-Mg^2+^ buffer (5 mM Tris, 1 mM EDTA pH 8.0). The liquid remaining in the dialysis unit was collected and analysed using AGE and negative-stain TEM.

### Nuclease degradation assay

Bare DNs, K_10_ or K_10_–PEG_5K_-coated DNs were diluted in RPMI-1640 medium (Gibco) containing different amounts of DNase I or 10% freshly thawed FBS (Gibco; heat-inactivated at 56 °C by the vendor) such that the final Mg^2+^ ion concentration is 0.6 mM. The samples were incubated at 37 °C for 0–24 h on a Tetrad 2 Peltier thermal cycler (Bio-Rad) and analysed using AGE and TEM imaging.

### Nuclease deactivation assay

Freshly prepared cell medium containing 10% FBS and solutions containing up to 50 U ml^−1^ DNase I could be inactivated by the addition of 5 mM EGTA and 10% β-mercaptoethanol, and incubation at 37 °C for 30 min.

### Removal of K_10_ or K_10_–PEG_*n*K_ shell from DNs

Chondroitin sulfate (100 × , excess to the number of amines) was added to K_10_ or K_10_–PEG_5K_-coated DNs and the medium adjusted to 10 mM Mg. The sample was incubated at 37 °C for 1–2 h during which the oligolysine shell was removed from the DNs upon electrostatic binding of oligolysine with chondroitin sulfate. In the nuclease degradation assays, nucleases were deactivated using EGTA and β-mercaptoethanol before the removal of the oligolysine shell.

### *In vitro* microscopy measurements

*Sample preparation*: a 1.5 coverslip and a glass slide were sandwiched together by two strips of double-sided tape to form a flow chamber. The volume of the flow chamber was ∼20 μl. The chamber was incubated for 30 min with 20 μl of 1 mg ml^−1^ BSA-Biotin, followed by incubation for 30 min with 20 μl of 1 mg ml^−1^ streptavidin. The chamber was then washed three times with PBS. Twenty microlitres of K_10_–PEG_5K_-coated biotinylated DNs were then introduced in the flow chamber at 10 nM concentrations. The chamber was epoxy sealed before imaging. *Imaging*: samples were imaged using Zeiss Axio Observer inverted fluorescence microscope. Samples were excited either with 540–580 nm LED (Colibri; Cy3 excitation) or with a 625 nm LED (Colibri; Cy5 excitation). Zeiss filter sets 43 HE and 50 were used for Cy3 and Cy5 imaging, respectively. Signal was collected using an Orca-Flash 4.0 sCMOS from Hamamatsu. All data were collected using a 10 × /0.3 Ph1 (WD=5.2 mm) Zeiss objective. Built-in image processing and analysis tools from Fiji (http://fiji.sc/Fiji) were used to analyse all data.

### Isolation and culture of BMDCs

BMDCs were derived using established methods^31^. Briefly, bone marrow cells were isolated from female C57Bl/6J mice (Jackson Laboratories) and cultured in RPMI (Lonza) supplemented with 10% heat-inactivated FBS (Sigma-Aldrich), 1% penicillin/streptomycin, 50 μM β-mercaptoethanol (Sigma-Aldrich) and 20 ng ml^−1^ granulocyte–macrophage colony-stimulating factor (Peprotech). Non-adherent dendritic cells between day 7 and 10 were harvested and used for experiments.

### Cell uptake assay

For uptake studies, BMDCs were seeded at a density of 50,000 cells per well into 1.5 glass-bottom 96-well plates from Mat-tek and allowed to grow overnight. Samples were prepared by diluting them to 10 nM concentrations in a total volume of 100 μl BMDC cell medium (RPMI-1640 supplemented with 10% FBS, β-mercaptoethanol and granulocyte–macrophage colony-stimulating factor). Cell uptake was monitored using confocal microscopy (SP5 × MP inverted confocal microscope, Leica) at time points between 0 and 24 h. A sequential *z*-stack was imaged using a 0.2 μm slice thickness, in the following excitation sequence: first scan: Cy3 excitation/Cy5 emission, second scan: bright field and Cy3 excitation/ Cy3 emission; and third scan: Cy5 excitation/Cy5 emission. To bleach the acceptor dye, region of interest was set to 2.5 × zoom of the initial field of view and laser power for Cy5 excitation was increased to 100%. The slice was bleached for 10 min, until the Cy5 signal was absent. Zoom was reset to 1 and a new *z*-stack of the initial field of view was recorded.

### Cell image analysis

*Z*-stacks were loaded into Imaris software package and dimensions were cropped to represent the cell area. The new stacks were imported into image processing and analysis software from Fiji and a *Z* projection based on average intensity was made. Regions inside and outside the bleached area were selected using the freehand selection tool and the integrated intensity was measured. DN**1** containing only 18 Cy3 (donor-only) dyes or 18 Cy5 dyes (acceptor only) were used to estimate the background corrections.

### Biodistribution experiments

Optical imaging experiments on mice were carried out using the IVIS spectrum instrument (Perkin Elmer). Three mice per sample were anaesthetized under 2% isofluorane and were injected retro-orbitally with 100 μl volume of Alexa 750 oligo, DN**1**_bare_ and DN**1**_coated_ functionalized with 36 Alexa 750 fluorophores such that final concentration of Alexa 750 fluorophore is 0.625 μM. The mice were immediately transferred to the imaging system and maintained at 2% isofluorane throughout imaging. Fluorescence images for kinetic analysis were acquired by a 30 s excitation at 745 nm and emission at 800 nm, every 2 min for a total of 1 h post injection. After collection of fluorescence data, the mice were immediately killed, and blood, urine and organs were harvested and were imaged on the IVIS system. Analysis was carried out on ImageJ. Fluorescence accumulation in the bladder was measured by using the freehand selection tool to draw a region of interest around the bladder and then integrating the density. The data set was normalized to the maximum fluorescence intensity in the bladder, when Alexa 750 dye oligo is administered alone. Fluorescence accumulation in bladder versus time was fitted to an exponential using MATLAB to extract half-life.

### Animal use

All animal studies were performed in accordance with NIH guidelines, under the approval of Harvard University's Institutional Animal Care and Use Committee.

### Data availability

Data supporting the findings of this study are available within the article (and its Supplementary Information files) and from the corresponding author upon reasonable request.

## Additional information

**How to cite this article:** Ponnuswamy, N. *et al*. Oligolysine-based coating protects DNA nanostructures from low-salt denaturation and nuclease degradation. *Nat. Commun.* **8,** 15654 doi: 10.1038/ncomms15654 (2017).

**Publisher's note**: Springer Nature remains neutral with regard to jurisdictional claims in published maps and institutional affiliations.

## Supplementary Material

Supplementary Information Supplementary Figures, Supplementary Tables and Supplementary References.

Supplementary Data 1 ponnuswamy_Supplementary_Data_1.xlsx: DNA sequences for DN1

Supplementary Data 2 ponnuswamy_Supplementary_Data_2.xlsx: DNA sequences for DN2

Supplementary Data 3 ponnuswamy_Supplementary_Data_3.xlsx: DNA sequences for DN3

Supplementary Data 4 ponnuswamy_Supplementary_Data_4.xlsx: DNA sequences for DN4

Supplementary 5 ponnuswamy_Supplementary_Data_5.xlsx: DNA sequences for DN5

Supplementary Data 6 ponnuswamy_Supplementary_Data_6.xlsx: DNA sequences for DN6

Supplementary Data 7 ponnuswamy_Supplementary_Data_7.xlsx: DNA sequences for DN7

Supplementary Data 8 ponnuswamy_Supplementary_Data_8.xlsx: DNA sequences for DN8

Supplementary Data 9 ponnuswamy_Supplementary_Data_9.xlsx: DNA sequences for DN9

Supplementary Data 10 ponnuswamy_Supplementary_Data_10.xlsx: DNA sequences for Cy3/Cy5functionalized DN1
