# Supplementary material for: Oligolysine-based coating protects DNA nanostructures from low-salt denaturation and nuclease degradation
Source: Nat Commun. 2017 May 31;8:15654. doi: 10.1038/ncomms15654 (PMC5460023; doi:10.1038/ncomms15654)
Supplement: Supplementary Information — Supplementary Figures, Supplementary Tables and Supplementary References. [file ncomms15654-s1.pdf]

Supplementary information for:

Ponnuswamy N et al. Oligolysine-based coating protects DNA nanostructures from low-salt denaturation and nuclease degradation

SUPPLEMENTARY FIGURES

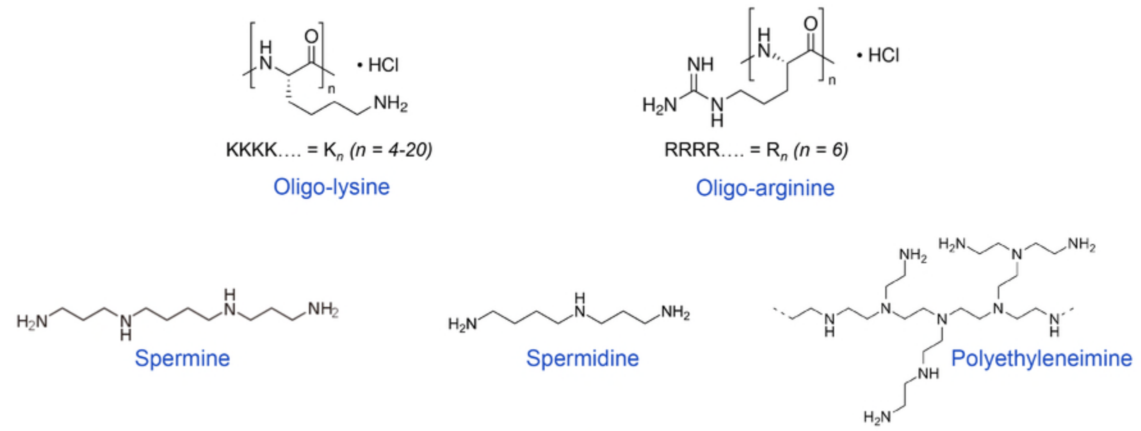

Supplementary Figure 1| Chemical structure of various polyamines used in this study.

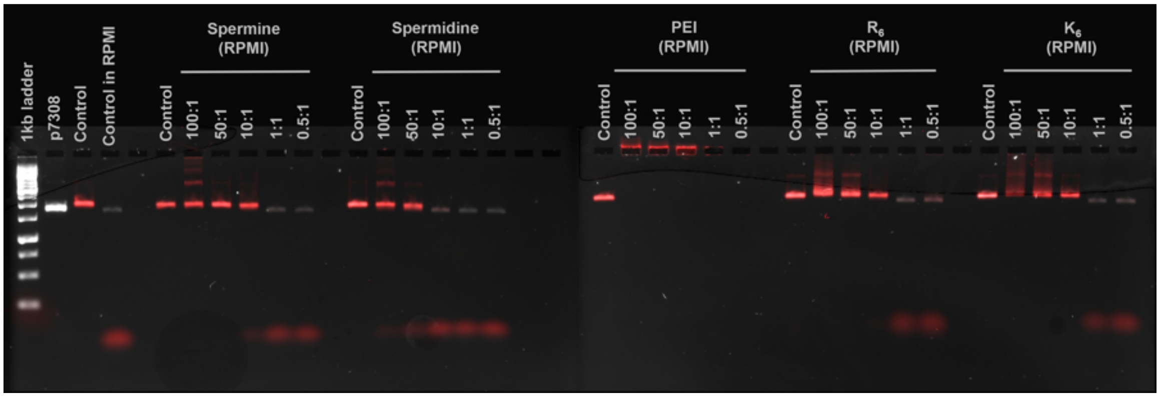

Supplementary Figure 2| Effect of different polyamines on the stability of DN1 in low-salt buffer at 37 °C. Cy5 labelled DN**1** (coloured red) was coated with different polyamines at various N:P ratios, depicted in the figure and then diluted with RPMI-1640 such that the overall Mg^{2+} concentration is 0.6 mM. The samples were incubated at 37 °C for 1 hour, then loaded onto a 2% agarose gel and ran in 0.5x TBE buffer containing 11 mM Mg^{2+} at 65 V for 2 hours. The sample labeled "control" is DN**1** in its folding conditions and the one labeled "control in RPMI' is when DN**1** is diluted in RPMI buffer. Without any coating the control structure is rapidly disassembled into constituent staples and scaffold as evidenced from the release of Cy5 labelled staples. Spermidine and spermine require very high N:P to stabilize the structures, at lower N:P release of staples is observed. PEI on the other hand condenses and crushes the structure at all N:P ratios tested. Oligoarginine (R_6) and oligolysine (K_6) show comparable stability profiles, however, coating with R_6 deforms the structures as observed by TEM.

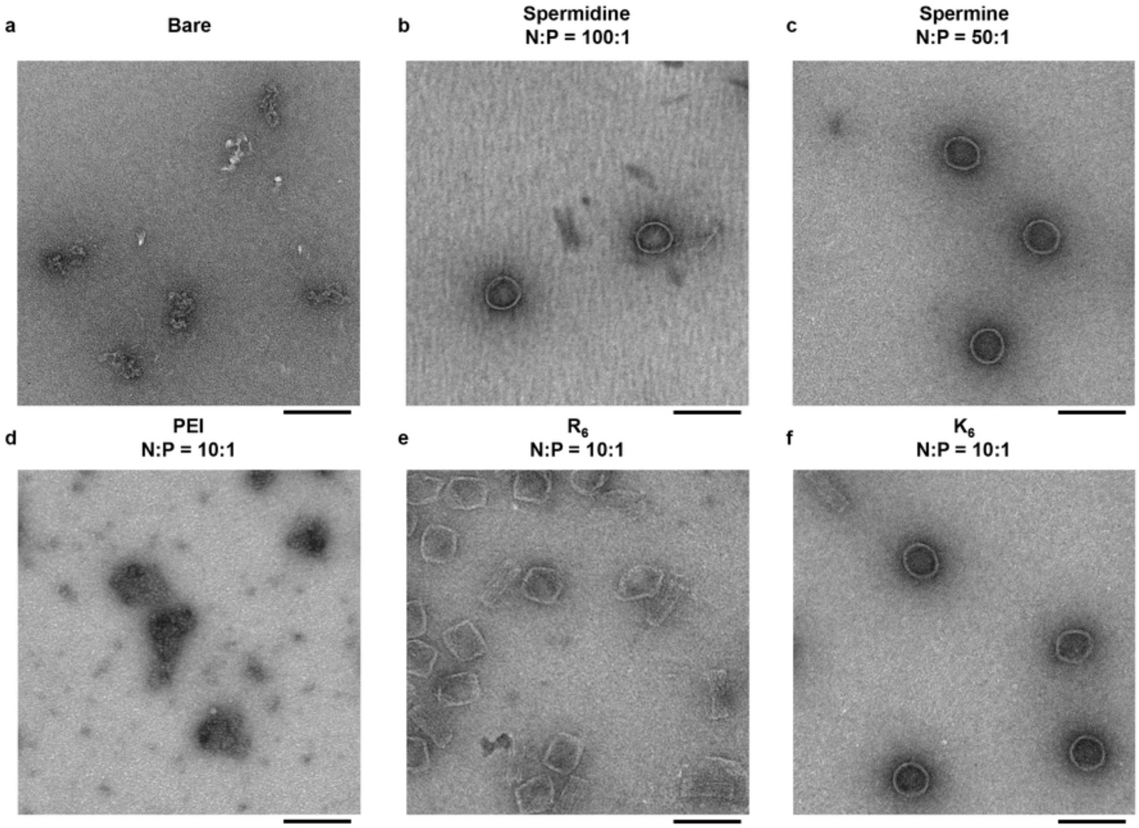

Supplementary Figure 3| Negative stain TEM images of DN1 coated with different polyamines in low-salt buffer at 37 °C. DN**1** was coated with different polyamines at various N:P ratios, depicted in the figure and then diluted with RPMI-1640 such that the overall Mg^{2+} concentration is 0.6 mM. The samples were incubated at 37 °C for 1 hour. a) Bare DN**1**; b) DN**1** + Spermidine (N:P = 100:1); c) DN**1** + Spermime (N:P = 50:1); d) DN**1** + PEI (N:P = 10:1); e) DN**1** + oligoarginine R_6 (N:P = 10:1); f) DN**1** + oligolysine K_6 (N:P = 10:1). Best results for stability in low-salt buffers were obtained for oligolysine K_6. DNs were stained using 2% uranyl formate for 30 seconds. Scale bars are 100 nm.

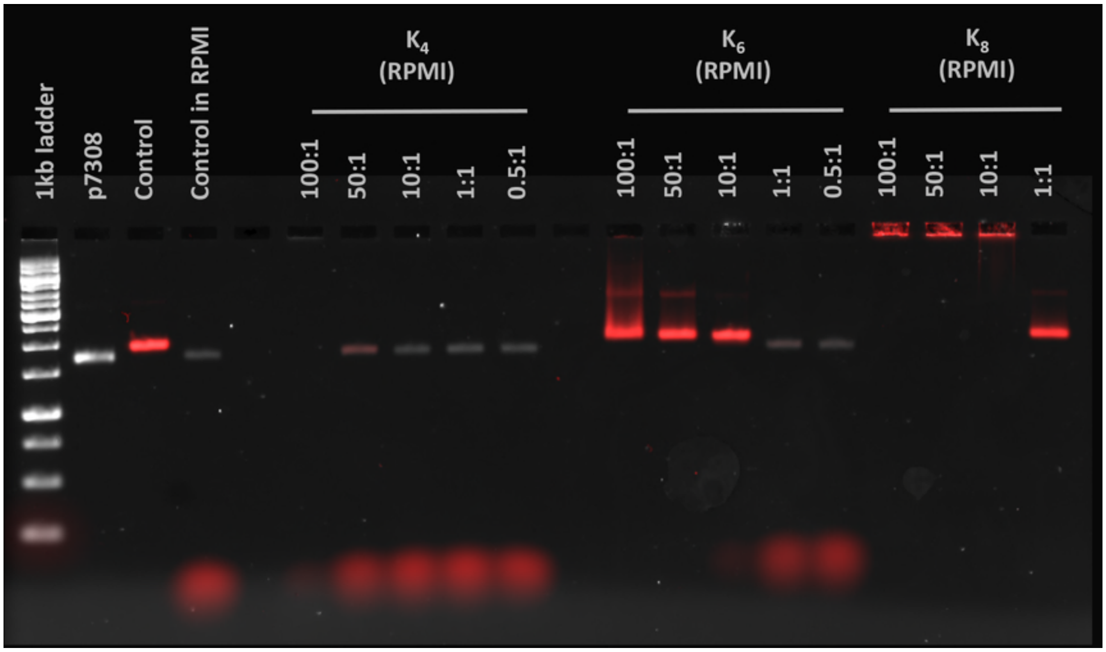

Supplementary Figure 4| Effect of different lengths of oligolysine on the stability of DN1 in low-salt buffer at 37 °C. Cy5 labelled DN**1** (coloured red) was coated with different lengths of oligolysine at various N:P ratios, depicted in the figure and then diluted with RPMI-1640 such that the overall Mg^{2+} concentration is 0.6 mM. The samples were incubated at 37 °C for 1 hour, then loaded onto a 2% agarose gel and ran in 0.5x TBE buffer containing 11 mM Mg^{2+} at 65 V for 2 hours. Longer lengths require lower N:P to stabilize the DNs and cause aggregation of DNs at higher N:P. Ethidium bromide staining is indicated as white contrast.

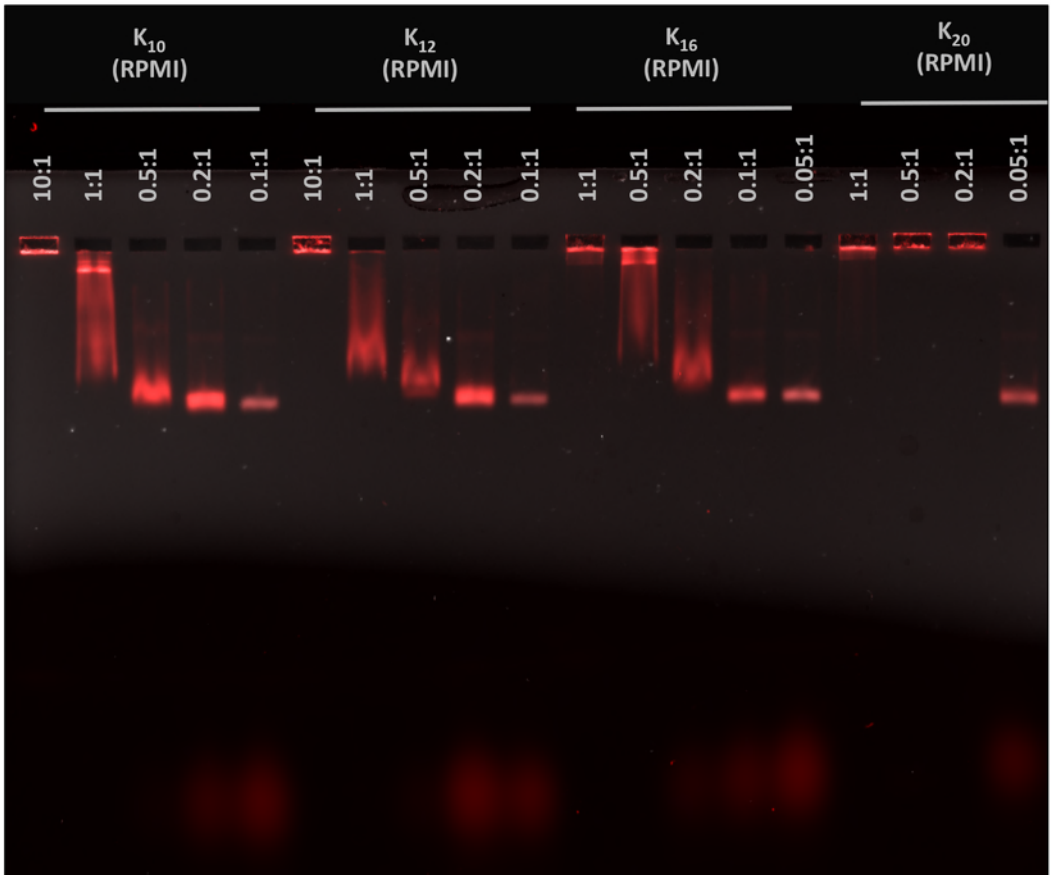

Supplementary Figure 5| Effect of different lengths of oligolysine on the stability of DN1 in low-salt buffer at 37 °C. Cy5 labelled DN**1** (coloured red) was coated with different lengths of oligolysine at various N:P ratios, depicted in the figure and then diluted with RPMI-1640 such that the overall Mg^{2+} concentration is 0.6 mM. The samples were incubated at 37 °C for 1 hour, then loaded onto a 2% agarose gel and ran in 0.5x TBE buffer containing 11 mM Mg^{2+} at 65 V for 2 hours. Longer lengths require lower N:P to stabilize the DNs and cause aggregation of DNs at higher N:P.

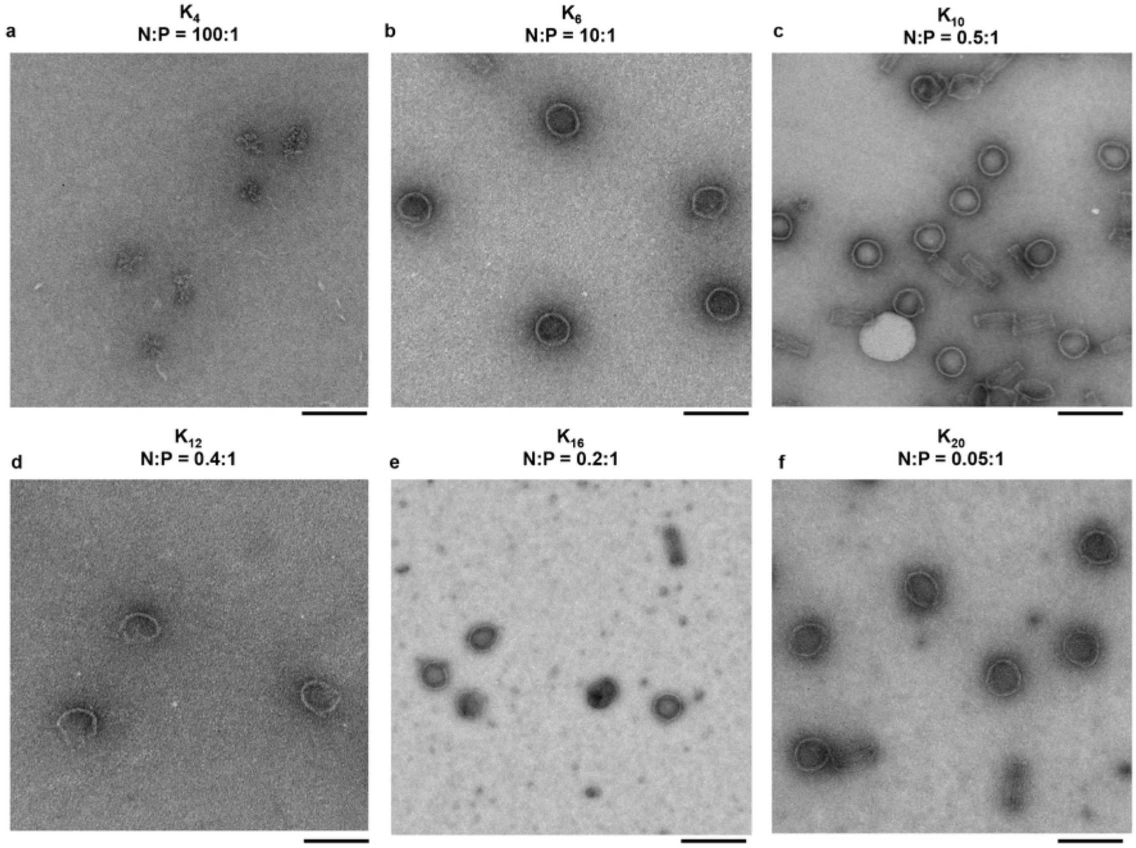

Supplementary Figure 6| Negative stain TEM images of DN1 coated with different lengths of oligolysine in low-salt buffer at 37 °C. DN**1** was coated with different polyamines at various N:P ratios, depicted in the figure and then diluted with RPMI-1640 such that the overall Mg^{2+} concentration is 0.6 mM. The samples were incubated at 37 °C for 1 hour. a) DN**1** + K_4 (100:1); b) DN**1** + K_6 (N:P = 10:1); c) DN**1** + K_{10} (N:P = 0.5:1); d) DN**1** + K_{12} (N:P = 0.4:1); e) DN**1** + K_{16} (N:P = 0.2:1); f) DN**1** + K_{20} (N:P = 0.05:1). Longer lengths require lower N:P to stabilize the DNs and cause aggregation of DNs at higher N:P. DNs were stained using 2% uranyl formate for 30 seconds. Scale bar is 100 nm in all images except 6e, where it is 200 nm.

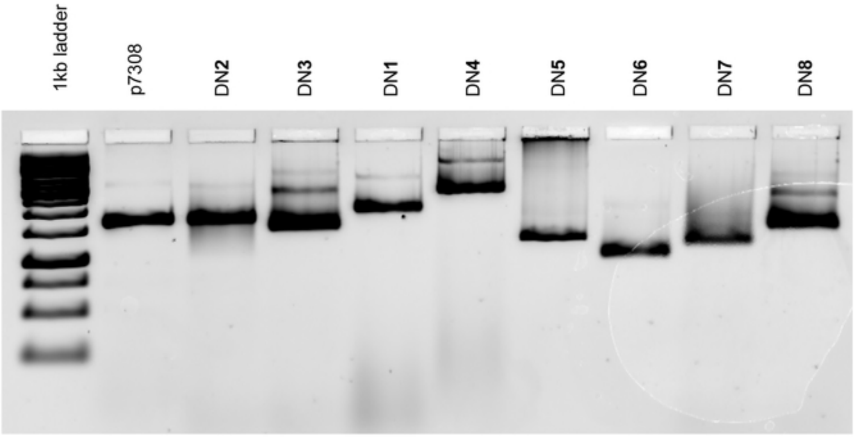

Supplementary Figure 7| AGE of different DNs. DN**1**–DN**8** were annealed as described in the methods and purified using the glycerol gradient ultracentrifugation method. 10µL of 10 nM purified samples were loaded onto a 2% agarose gel and ran in a 0.5x TBE buffer containing 11 mM Mg^{2+} at 65 V for 2 hours.

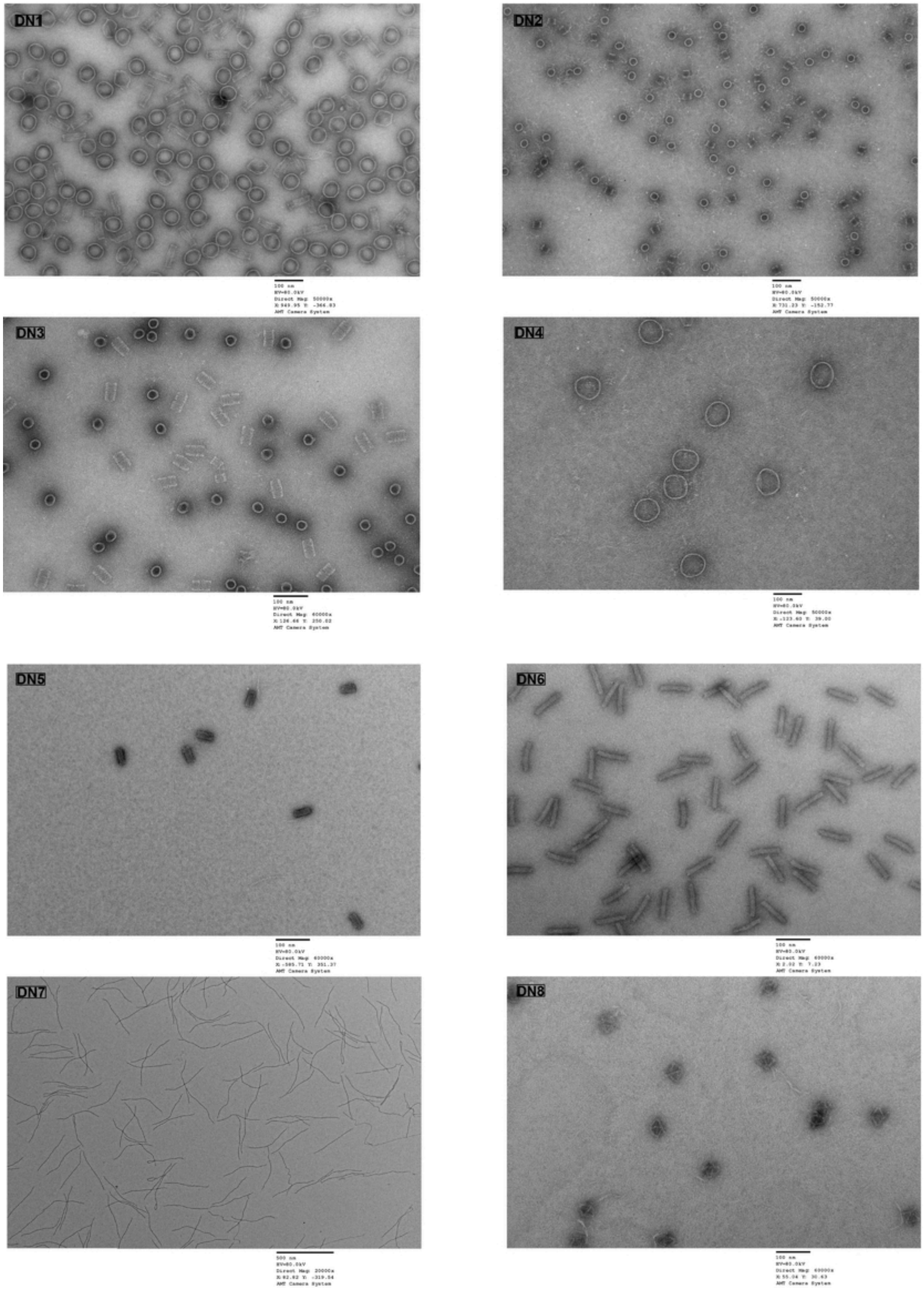

Supplementary Figure 8| Negative stain TEM images of purified DN1-DN8. DNs were stained using 2% uranyl formate. Scale bars are 500 nm for DN7 and 100 nm for all other panels.

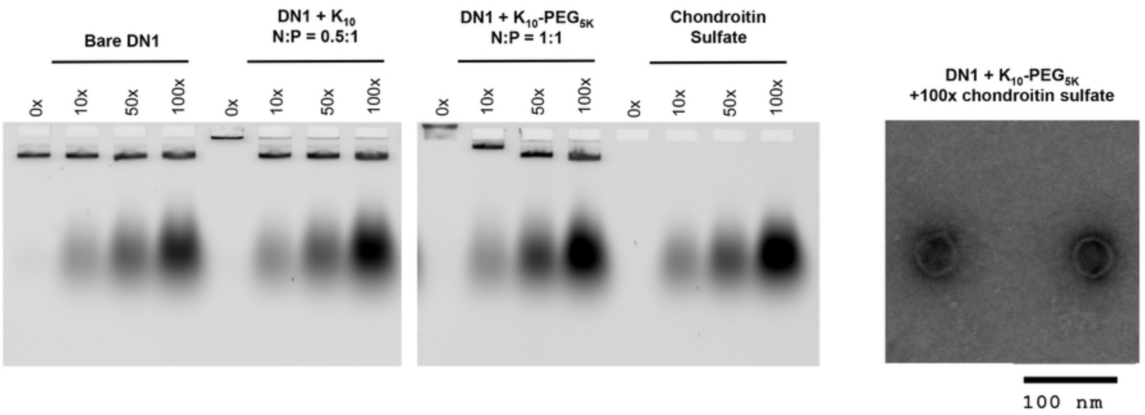

Supplementary Figure 9| Chondroitin sulfate removes oligolysine coating from DNs without damaging the structures. This is a useful operation, as it allows gel analysis of oligolysine-coated DN structures that have been subjected to some environmental condition while N:P = 1; without stripping the oligolysine, such structures have neutral charge and won't enter the gel. Bare, K_{10} coated and K_{10}-PEG_{5K} coated DN**1** were incubated with different excess of chondroitin sulfate relative to the amount of amines in oligolysine for 2 hours at 37 °C. The Mg^{2+} concentration was adjusted to 10 mM. The samples were loaded on a 2% agarose gel and ran in 0.5x TBE buffer containing 11 mM Mg^{2+} at 65 V for 1 hour. Intact structures were observed under negative stain TEM for DN**1** when incubated with 100x chondroitin sulfate. Note that DN**1** has been purified of excess staples using glycerol gradient and that chondroitin sulfate is stained by ethidium bromide. Scale bar is 100 nm.

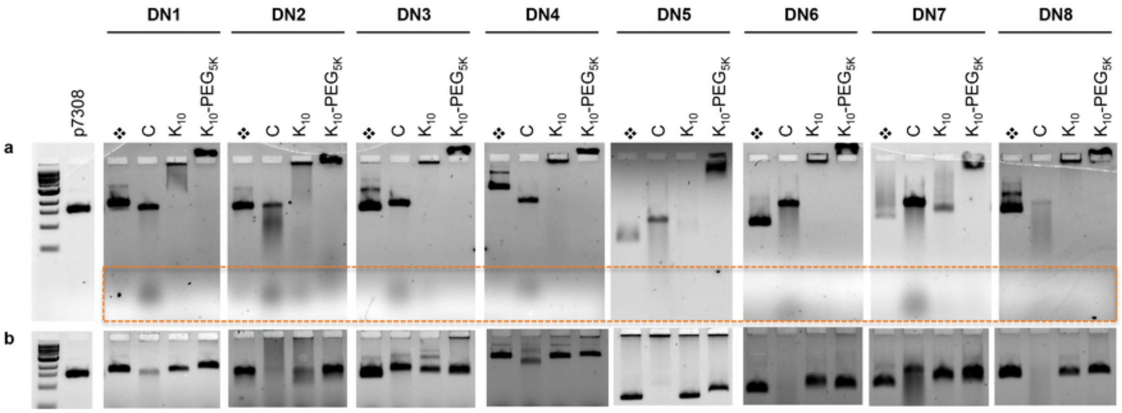

Supplementary Figure 10| Dialysis of DN1-DN8 into zero-Mg^{2+} buffer (5mM Tris, 1mM EDTA, pH 7). 50µL of bare, K_{10} coated and K_{10}-PEG_{5k} coated DN**1**-DN**8** were dialyzed into 1L of zero-Mg^{2+} buffer overnight at room temperature. '❖' refers to an undialyzed positive control and 'C' refers to the dialyzed bare DNs. **a)** An aliquot of the undialyzed positive control and the dialyzed samples were run on a 2% agarose gel in 0.5x TBE containing 11 mM Mg^{2+} for 3 h at 65 V. The release of the constituent staple strands can be observed upon dialysis for bare DNs. **b)** An aliquot of the undialyzed positive control and the dialyzed samples were first incubated with 50 mM chondroitin sulfate (at 100x amine concentration) at 37 °C for 3 hours to remove the oligolysie shell (Supplementary Fig. 9) and then ran on a 2% agarose gel in 0.5x TBE containing 11 mM Mg^{2+} for 3 h at 65 V. Structural integrity of DNs was preserved when they were coated with K_{10} and K_{10}-PEG_{5K}.

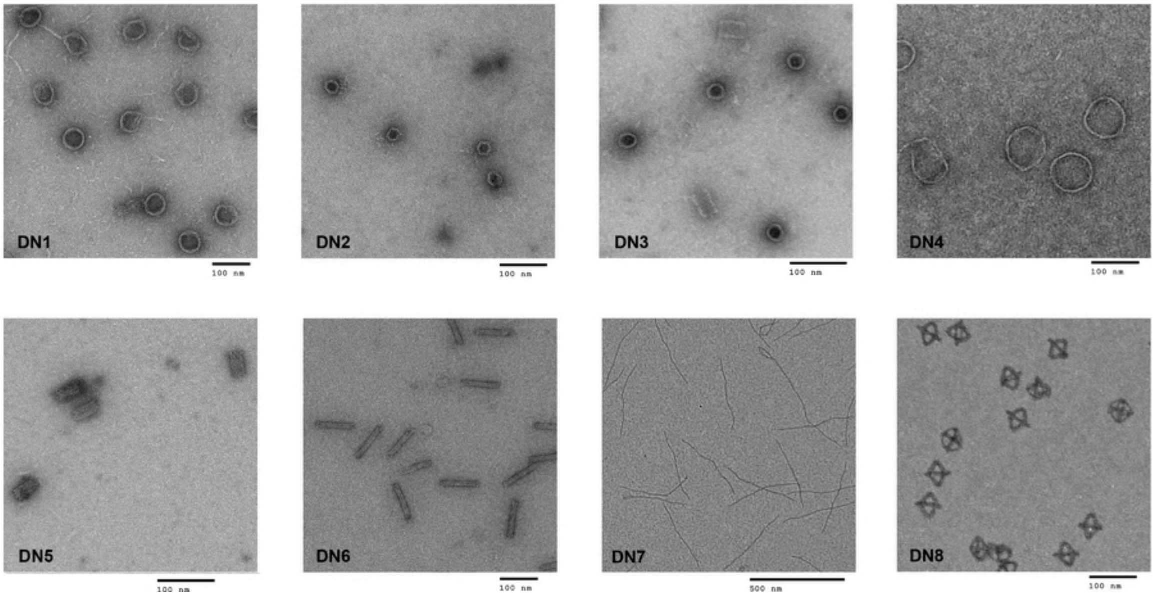

Supplementary Figure 11| TEM of K_{10} coated DNs dialyzed in zero-Mg^{2+} buffer (5mM Tris, 1mM EDTA, pH 7). Negative stain TEM images of K_{10} coated DN**1**-DN**8** after being dialyzed into zero-Mg^{2+} buffer overnight at room temperature. Scale bars are 500 nm for DN7 and 100 nm for all other panels.

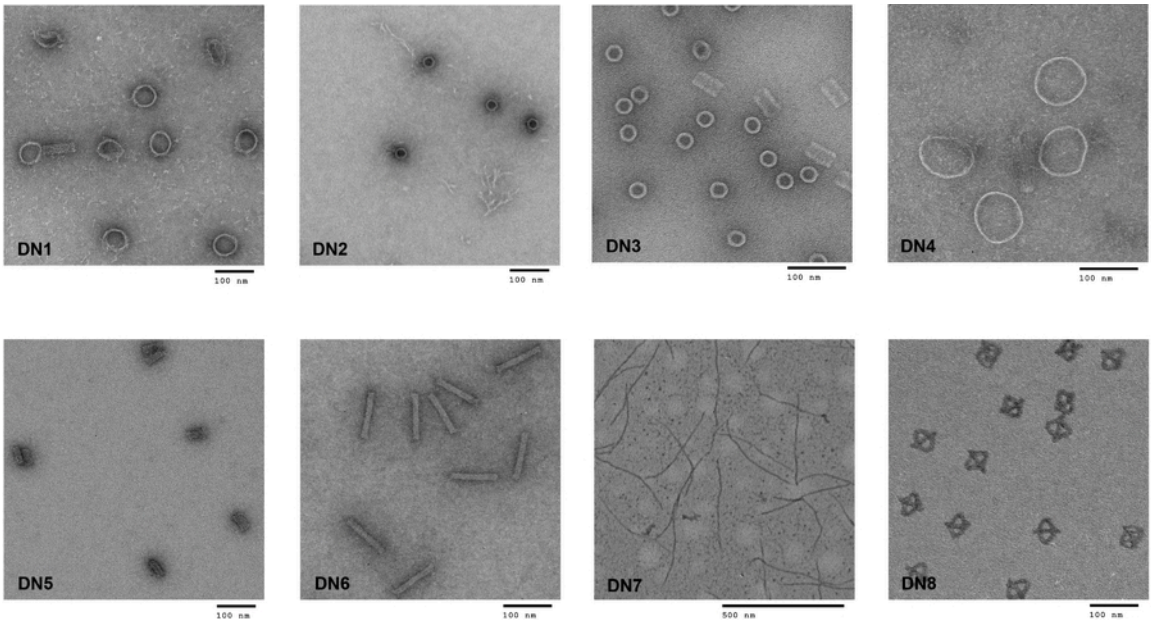

Supplementary Figure 12| TEM of K_{10}-PEG_{5K} coated DNs dialyzed in zero-Mg^{2+} buffer (5mM Tris, 1mM EDTA, pH 7). Negative stain TEM images of K_{10}-PEG_{5K} coated DN**1**-DN**8** after being dialyzed into zero-Mg^{2+} buffer overnight at room temperature. Scale bars are 500 nm for DN7 and 100 nm for all other panels.

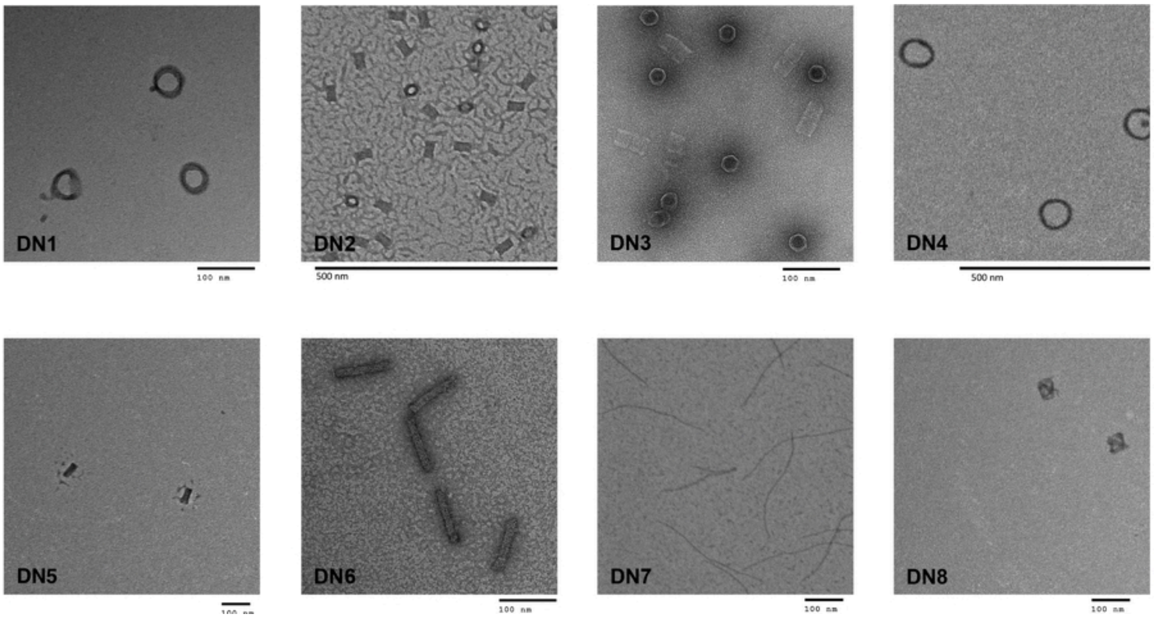

Supplementary Figure 13| TEM of K_{10}-PEG_{5K} coated DNs dialyzed in distilled water. Negative stain TEM images of K_{10}-PEG_{5K} coated DN**1**-DN**8** after being dialyzed into distilled water overnight at room temperature. Scale bars are 500 nm for DN2 and DN4 and 100 nm for all other panels.

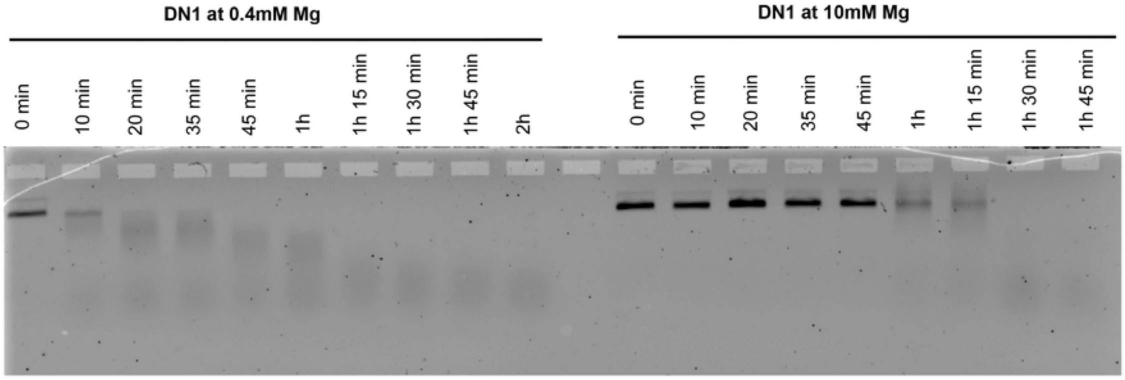

Supplementary Figure 14| Nuclease degradation of DN1 by DNase I in RPMI-1640 at 37 °C is dependent on the overall Mg^{2+} concentration. DN**1** (final concentration 1 nM) was incubated with 1 U/mL of DNase I in RPMI-1640 buffer at 37 °C for different time points. RPMI-1640 buffer was either used as it is (contains 0.4 mM Mg) or was supplemented with additional Mg^{2+} such that the final concentration is 10 mM. Incubated samples were then loaded on a 2% agarose gel and ran in 0.5x TBE buffer containing 11 mM Mg^{2+} at 65 V for 2 hours. Gel intensity can be used as a measure for structural degradation. We observe that nuclease degradation of DN**1** by DNase 1 is Mg^{2+} dependent. We hypothesize this is primarily due to the preservation of overall structure at higher Mg^{2+} concentrations, and that low-salt denatured structures are much better substrates for serum nucleases, perhaps due to their more open and accessible configurations.

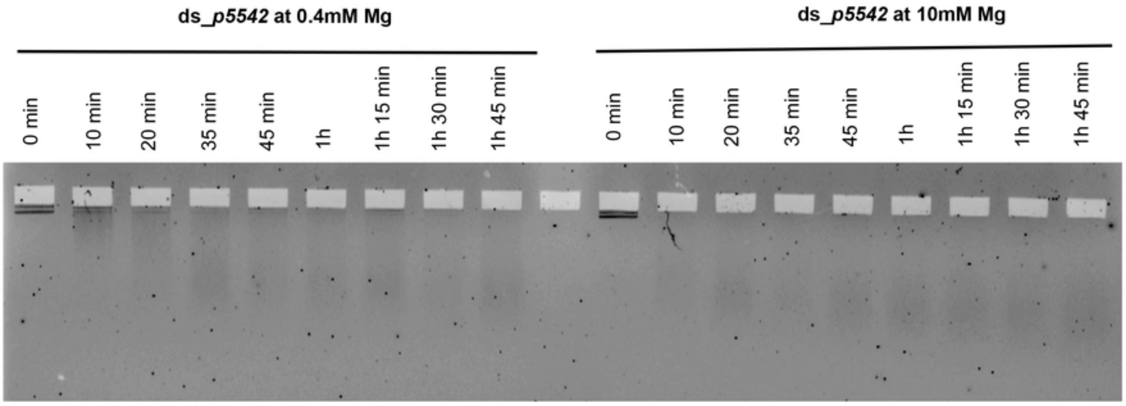

Supplementary Figure 15| Nuclease degradation of plasmid ds DNA (p5542) by DNase 1 in RPMI-1640 at 37 °C is independent of the overall Mg^{2+} concentration. Plasmid p5542 (final concentration 1 nM) was incubated with 1 U /mL of DNase I in RPMI-1640 buffer at 37 °C for different time points. RPMI-1640 buffer was either used as it is (contains 0.4 mM Mg) or was supplemented with additional Mg^{2+} such that the final concentration is 10 mM. Incubated samples were then loaded on a 2% agarose gel and ran in 0.5x TBE buffer containing 11 mM Mg at 65 V for 2 hours. Gel intensity can be used as a measure for structural degradation. In contrast to DN**1**, no effect of magnesium on the rate of degradation of p5542 was observed.

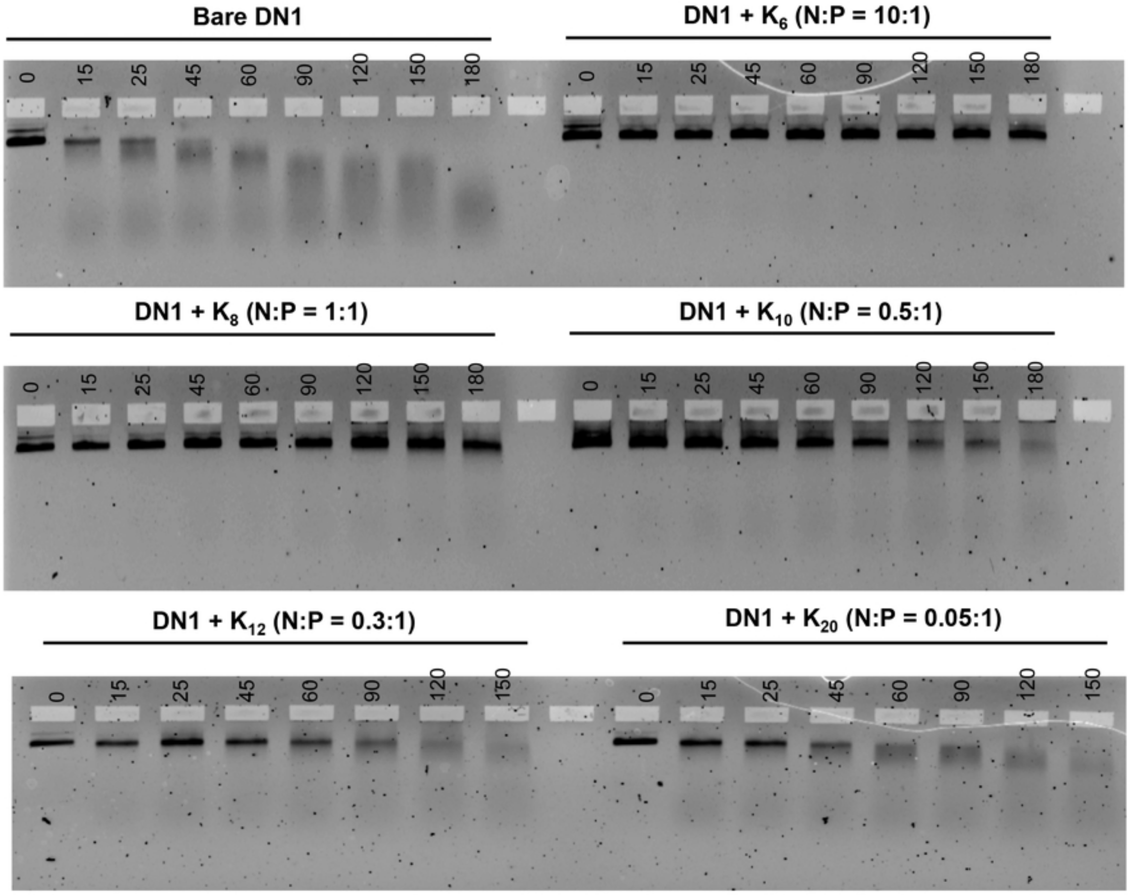

Supplementary Figure 16| Effect of length of oligolysine on the DNase I degradation of DN1. DN1 (final concentration 1 nM) coated with different oligolysines at N:P ratios depicted in the figure was incubated with 1 U/mL of DNase I in RPMI-1640 buffer at 37 °C for different time points. Incubated samples were then loaded on a 2% agarose gel and ran in 0.5x TBE buffer containing 11 mM Mg at 65 V for 2 hours. Gel intensity can be used as a measure for structural degradation. Higher N:P of oligolysine seems to offer better protection against nuclease degradation. With longer oligolysines, higher N:P can not be afforded as they lead to aggregation of the nanostructures.

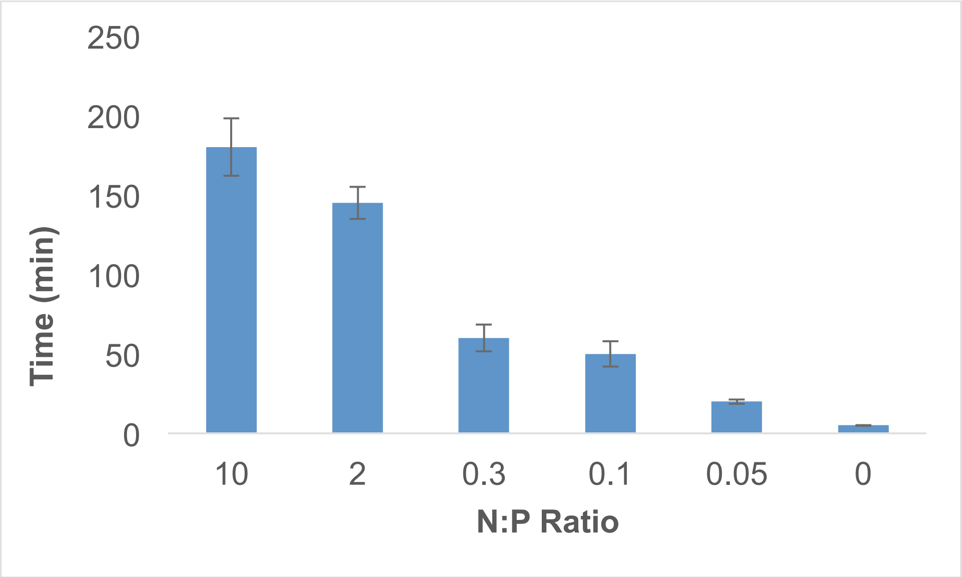

Supplementary Figure 17| Effect of N:P ratio of the oligolysine on the DNase I degradation of DN1. DN**1** (final concentration 1 nM) coated with different oligolysines at N:P ratios depicted in the figure was incubated with 1 U/mL of DNase I in RPMI-1640 buffer at 37 °C for different time points. A plot of $t_{1/2}$ (50% degradation as visualised by gel intensity) vs N:P ratio. Higher N:P of the oligolysine offers higher protection against nuclease degradation. Error bars represent standard deviation (n=3).

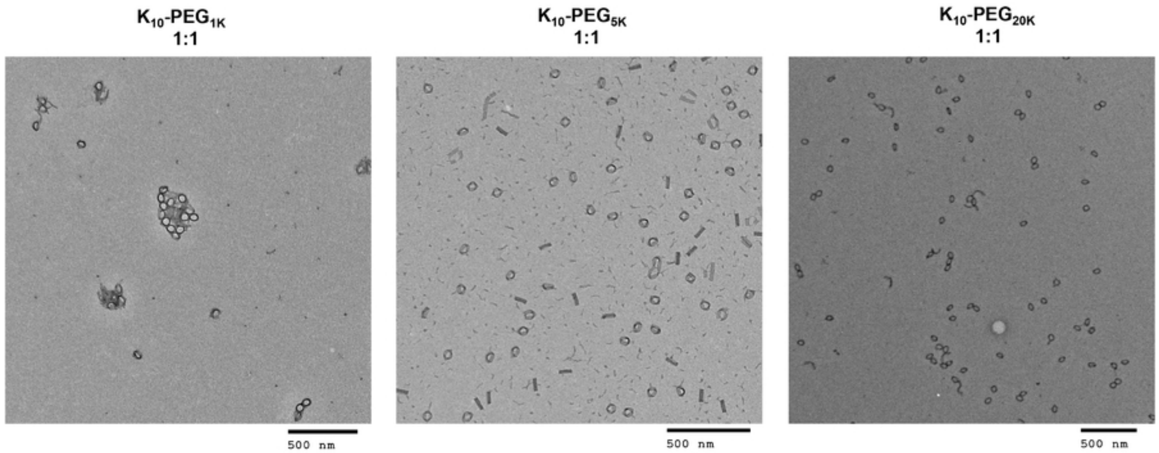

Supplementary Figure 18| Effect of size of conjugated PEG in K_{10}-PEG_{nK} on aggregation of structures. DN**1** was coated with 1:1 N:P ratio of K_{10}-$PEG_{1K\ /\ 5K\ or\ 20K}$ and imaged on TEM using negative stain to check for aggregation. Scale bars are 500 nm.

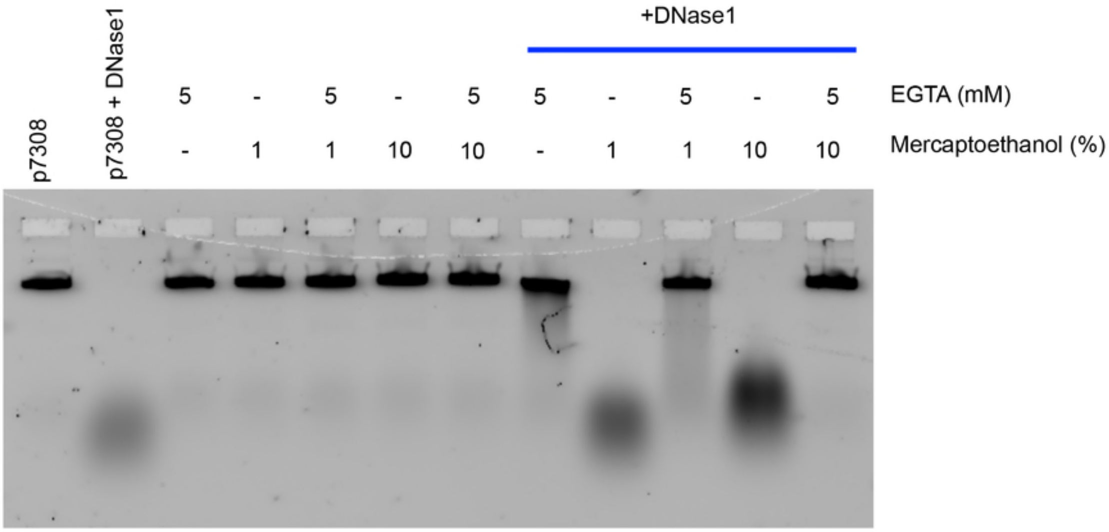

Supplementary Figure 19| Deactivation of DNase I by EGTA and β-mercaptoethanol in RPMI-1640 at 37 °C. p7308 (final conc 2 nM) was incubated with 50U/mL DNase I in RPMI-1640 at 37 °C for 1 hour. Different amounts of EGTA (ethylene glycol-bis(β-aminoethyl ether)-N,N,N',N'-tetraacetic acid) and β-mercaptoethanol were added to the above samples to deactivate DNase I and prevent degradation of p7308 scaffold. The best deactivation of DNase I was observed with 5 mM EGTA and 10% β-mercaptoethanol.

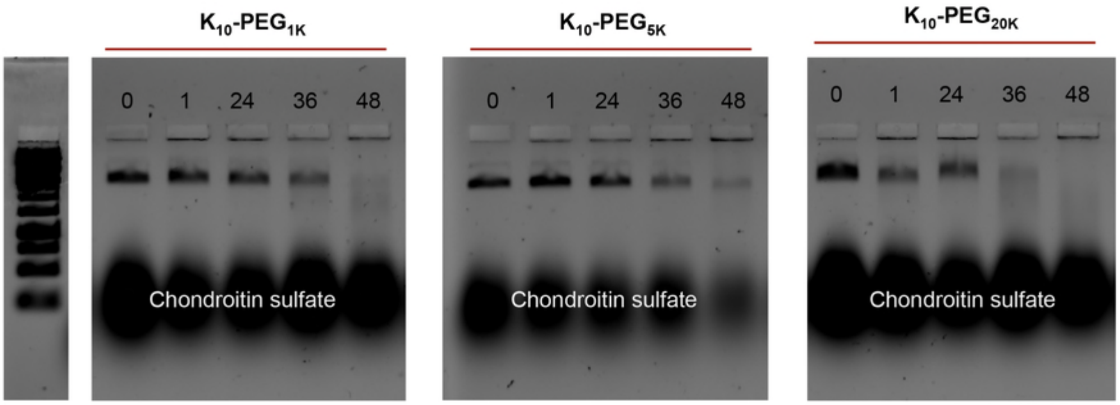

Supplementary Figure 20| Effect of size of conjugated PEG in K_{10}-PEG_{nK} on the nuclease degradation of DN1 in fresh culture medium at 37 °C. The stability of DN**1** coated with K_{10}-$PEG_{1K\ /\ 5K\ or\ 20K}$ was tested. Samples were incubated with freshly prepared BMDC culture medium (RPMI-1640 supplemented with 10% FBS, 2-mercaptoethanol and GM-CSF) for time intervals between 0h – 48h and ran on a 2% agarose gel in 0.5x TBE buffer containing 11 mM Mg^{2+}. Note that the nucleases were first deactivated using 5 mM EGTA and 10% β-mercaptoethanol and then the samples were incubated with 100x chondroitin sulfate prior to agarose gel to remove the PEG shell. All the PEG shells offer comparable stability towards nuclease degradation.

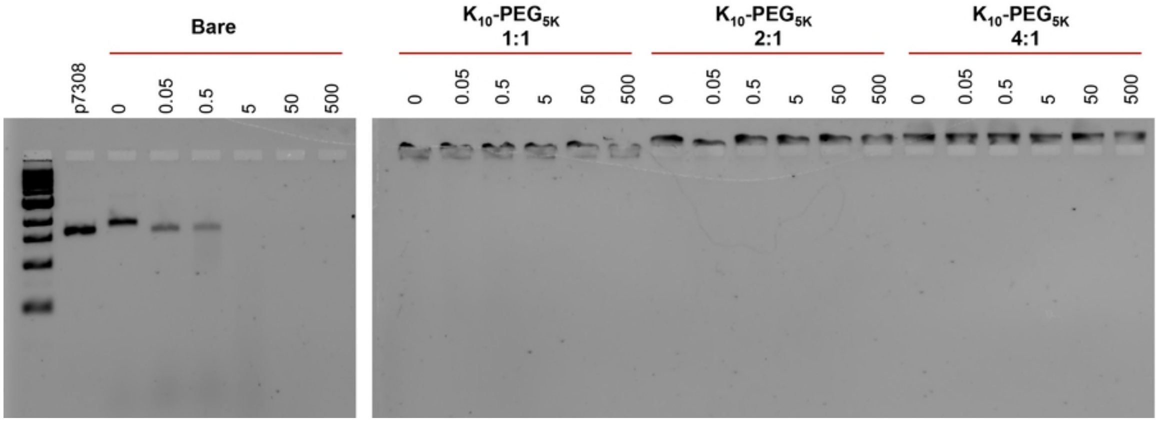

Supplementary Figure 21| Effect of K_{10}-PEG_{5K} coating on the DNase I degradation of DN1 in RPMI-1640 at 37 °C. Bare DN**1** (final concentration 1 nM) and DN**1** coated with K_{10}-PEG_{5K} at N:P of 1:1, 2:1 and 4:1 was incubated with different U/mL of DNase I in RPMI-1640 buffer at 37 °C. Incubated samples were then loaded on a 2% agarose gel and ran in 0.5x TBE buffer containing 11 mM Mg^{2+} at 65 V for 2 hours. Note that the PEG shells have not been removed in this experiment, as the DNAse I above 50U/mL could not be fully deactivated. Gel intensity can be used as a measure for structural degradation. All the PEG N:P ratios tested offer comparable stability towards DNase I degradation.

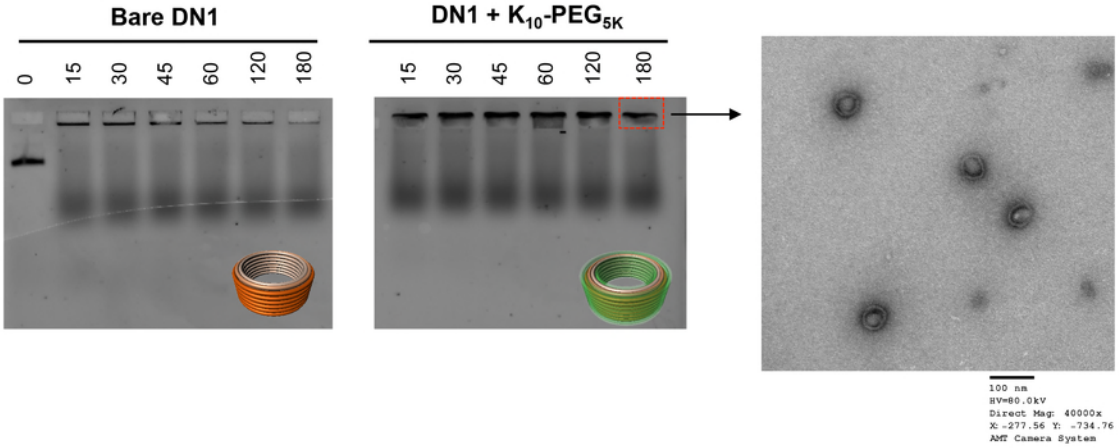

Supplementary Figure 22| Comparison of nuclease degradation of bare and K_{10}-PEG_{5K} coated DN1 in mouse serum at 37 °C. DN**1** (final concentration 1 nM) coated with K_{10}-PEG_{5K} at N:P of 1:1 was incubated with mouse serum at 37 °C Time points are shown in minutes. Note that the PEG shells have not been removed in this experiment, as the mouse serum could not be fully deactivated. Gel intensity can be used as a measure for structural degradation. While the bare DN**1** degraded very rapidly, K_{10}-PEG_{5K} coated DN**1** maintains structural integrity until the time tested, i.e. 3 hours. Scale bar is 100 nm.

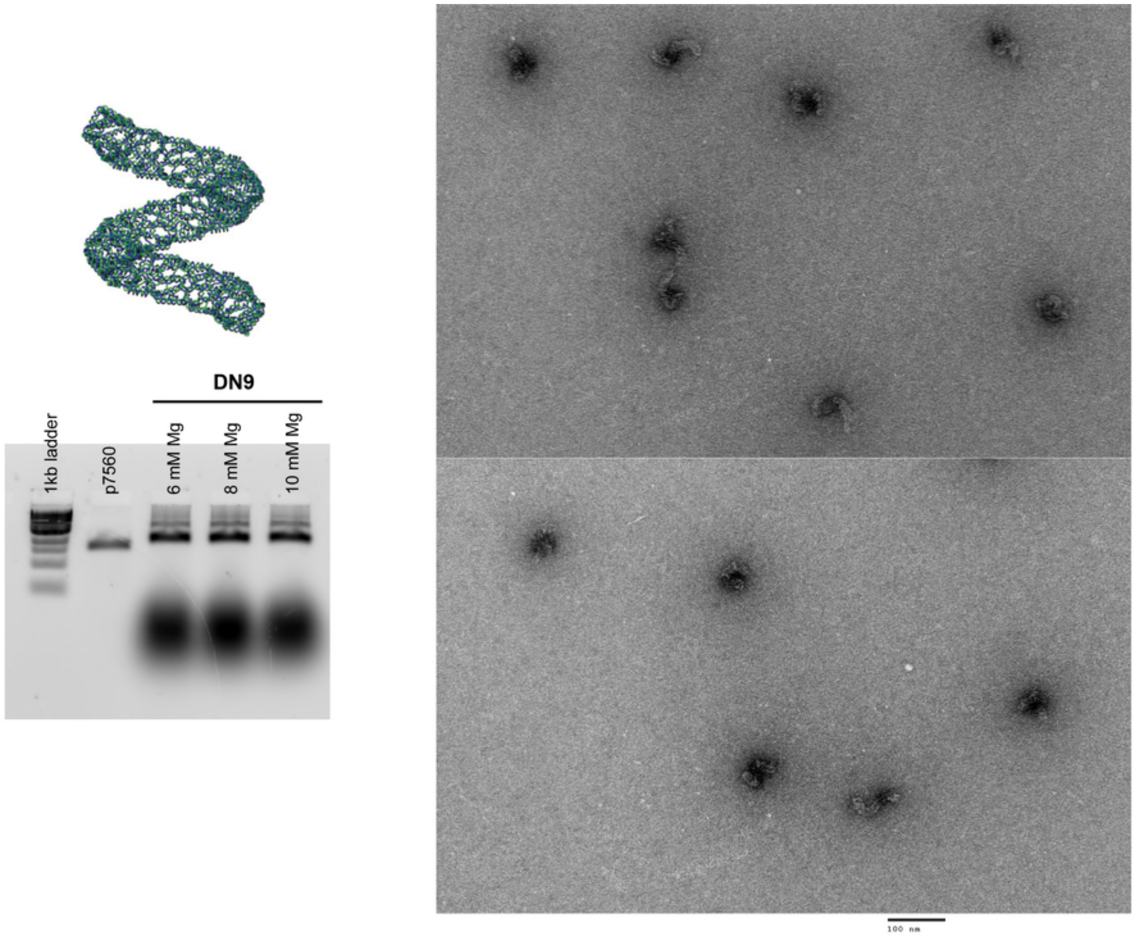

Supplementary Figure 23| AGE and TEM characterization of DN9. DN**9** was annealed using the protocol described in the methods. Three Mg^{2+} concentrations (6mM, 8 mM, 10 mM) were tested during annealing, all of which gave comparable yields. 10μL of 10 nM crude samples were loaded onto a 2% agarose gel and ran in a 0.5x TBE buffer containing 11 mM Mg^{2+} at 65 V for 2 hours. All the Mg concentrations tested show comparable folding yield. Negative stain TEM of DN**9** showed expected helical structures. The structures were stained with 2% uranyl formate. Scale bar is 100 nm.

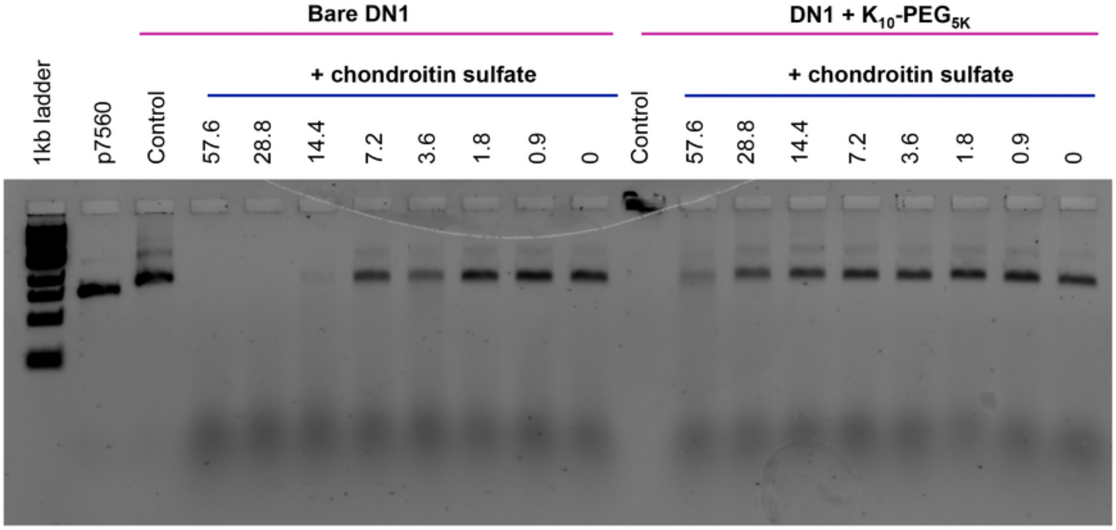

Supplementary Figure 24| Stability of K_{10}-PEG_{5K} coated DN9 against DNase1 degradation in RPMI-1640 at 37 °C. Bare DN**9** (final concentration 1 nM) and DN**9** coated with K_{10}-PEG_{5K} at N:P of 1:1 was incubated with different U/mL of DNase I in RPMI-1640 buffer at 37 °C. Incubated samples were then loaded on a 2% agarose gel and ran in 0.5x TBE buffer containing 11 mM Mg^{2+} at 65 V for 2 hours. Note that the DNase I was first deactivated using 5 mM EGTA and 10% β-mercaptoethanol and then the samples were incubated with 100x chondroitin sulfate prior to agarose gel to remove the PEG shell. DN**9** exhibited ~five fold greater nuclease resistance when coated with K_{10}-PEG_{5K}. This result suggests that the nuclease resistance offered by K_{10}-PEG_{5K} is in part due to steric shielding provided by the PEG layer that hinders binding of nucleases to the DNA.

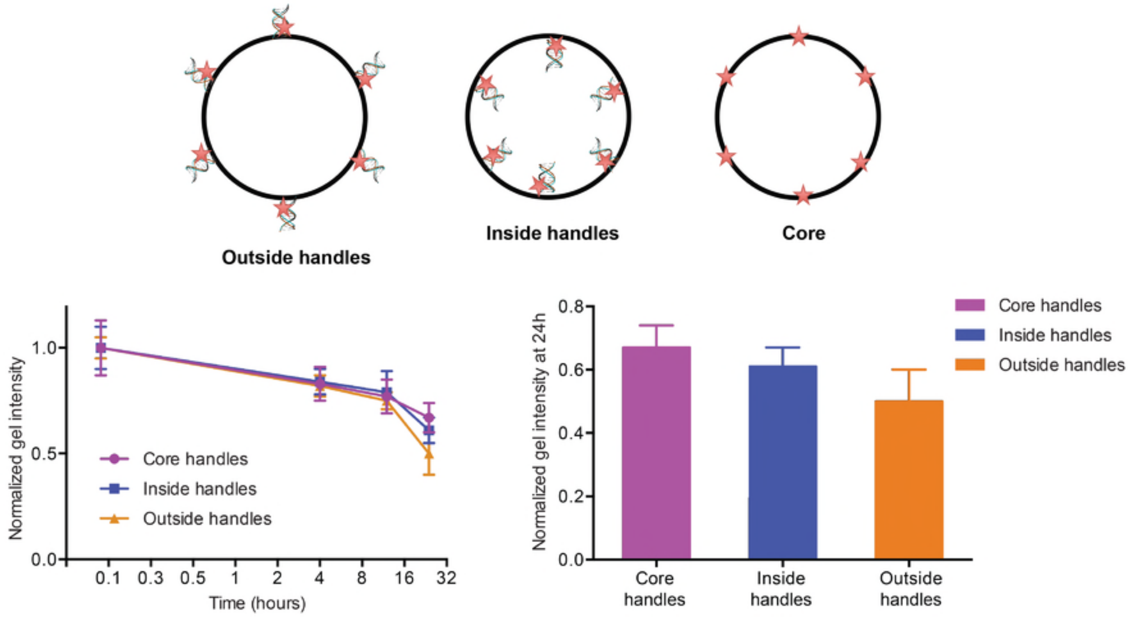

Supplementary Figure 25| Stability of handles on DN1 towards nuclease degradation. The stability of handles DN**1** was tested by placing the handles either on the outside or on the inside or at the core of the DN**1** nanostructure. For the outside and inside handles, we incorporated either twelve 21mer ssDNA handles on the outside of the DN**1** or twelve 21mer ssDNA handles on the inside of the DN**1**, to be bound by Cy5 labelled DNA anti-handles recruited from bulk solution. For the core handles, the 5´ of the inner staple oligos of DN**1** were directly functionalized with Cy5 fluorophore. All the above DN**1** samples were then coated with K_{10}-PEG_{5K} at N:P of 1:1. Samples were incubated with freshly prepared BMDC culture medium for time intervals between 0h – 24h and ran on a 2% agarose gel in 0.5x TBE buffer containing 11 mM Mg^{2+}. Measurable differences in degradation are only observed at the 24h time point. The handles at the core of DN**1** are the most stable followed by handles placed on the inside and finally handles placed on the outside. Error bars represent standard deviation (n=2).

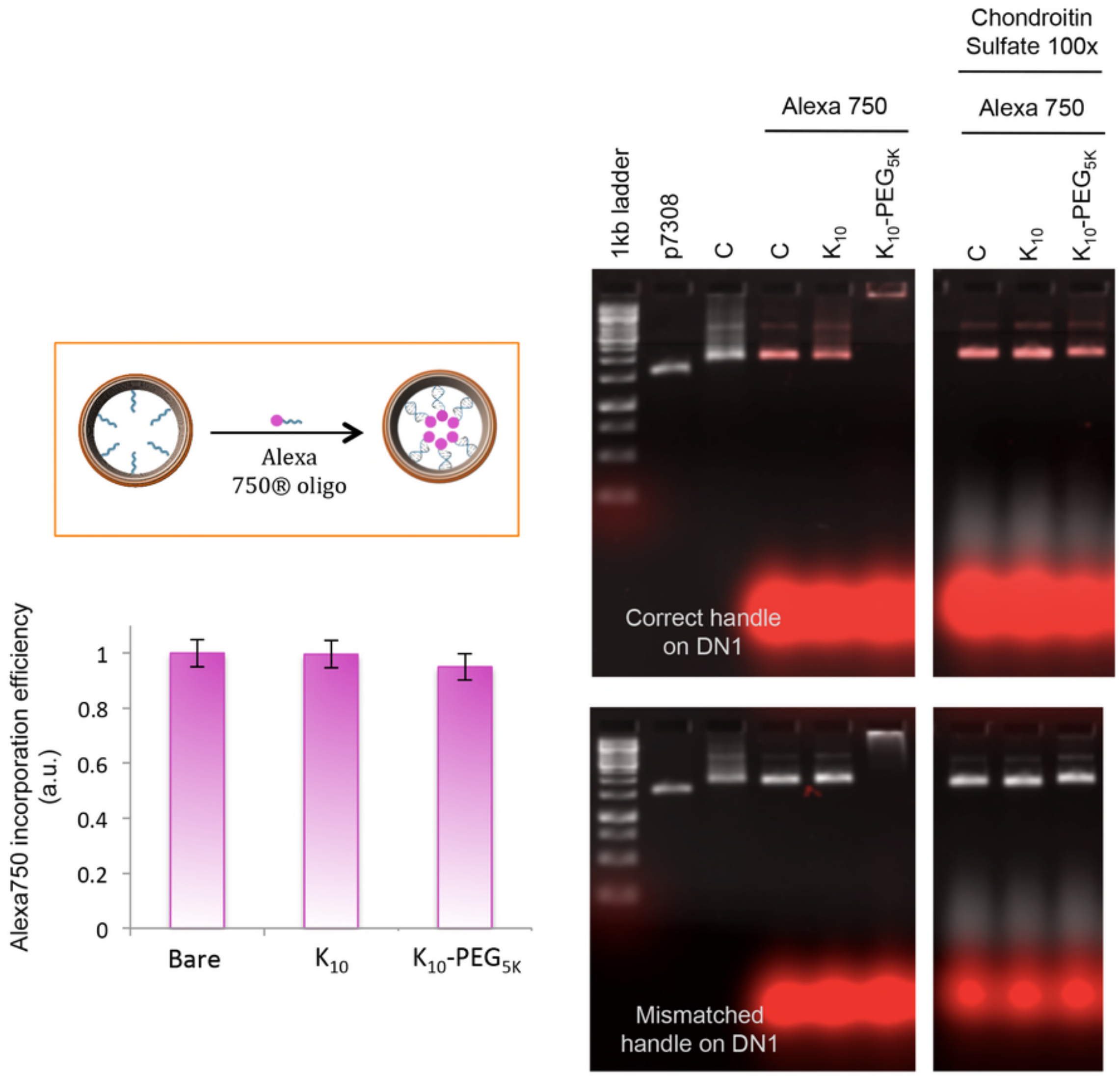

Supplementary Figure 26| Oligolysine coating of DNs does not prevent accessibility of ss DNA handles. We assayed the ability of ssDNA handles protruding of the surface of DNs to interact with ssDNA anti-handles in solution. For this purpose, we incorporated 12 inward-facing 21mer ssDNA handles on the inside of DN**1**, to be bound by Alexa-750 labelled DNA anti-handles recruited from bulk solution. We incubated DN**1** containing the ssDNA handles, bare versus K_{10} coated versus K_{10}-PEG_{5K} coated, with Alexa-750 labelled DNA anti-handle for 2 hours at 37ºC. The oligolysine coating was removed using 100x chondroitin sulfate to recover the original origami mobility. The samples were then loaded on a 2% agarose gel ran in 0.5x TBE buffer containing 11 mM Mg^{2+} at 65 V for 2 hours. Intensity of the Alexa channel (red) was then compared for the 3 variants of DN**1** and incorporation efficiency was calculated relative to the bare DN**1**. As a control to non-specific adsorption of the Alexa-750 on the DNA surface, DN**1** with mismatched handle was also included as a negative control in the experiment. No Alexa-750 was incorporated when mismatched handles were used. Error bars represent standard deviation (n=2).

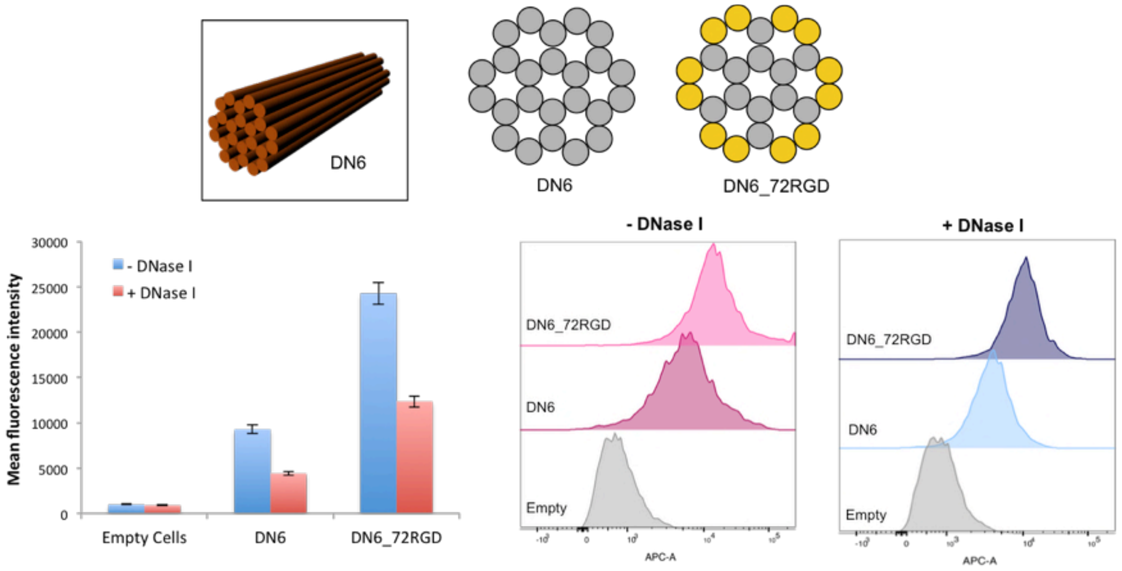

Supplementary Figure 27 | Uptake of DN6 into HUVE cells, monitored by FACS, as a function of cRGD ligands. Each helix in DN**6** that is coloured in yellow is labelled with 6 cRGD ligands using handle-antihandle binding. Left) Normalized fluorescence intensities of HUVE cells after 12 hour incubation with 1 nM Cy5-labeled DN**6** with and without DNase I treatment. Blue bars represent cells untreated by DNase I (i.e. internally + externally associated particles) and red bars represent cells treated by 40 U of DNase I (i.e. internally associated particles only). Error bars represent standard deviation (n=3). Centre) Fluorescence distribution for HUVE cells incubated with 1 nM Cy5-labeled DN6 untreated by DNase I as measured by FACS. Right) Fluorescence distribution for HUVE cells incubation with 1 nM Cy5 labelled DN6 treated by 40 U of DNase I as measured by FACS.

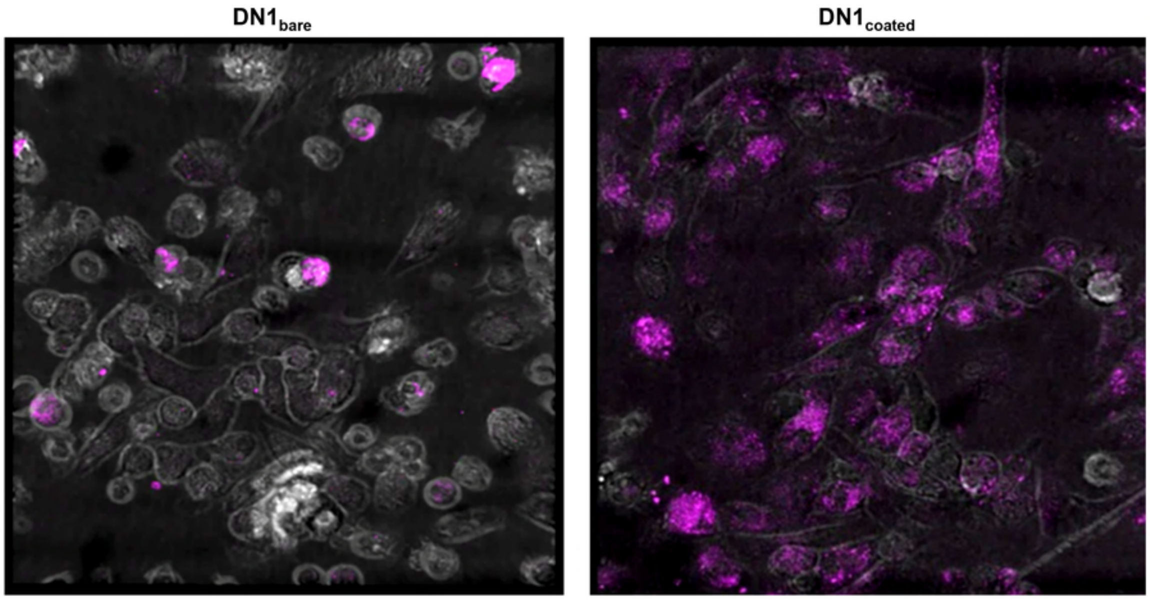

Supplementary Figure 28| K_{10}-PEG_{5K} coated structures are taken up by BMDC and the coating is non-toxic. Left: Bare and **Right:** K_{10}-PEG_{5K} coated Cy5 labelled DN**1** (5 nM, pink) was incubated with Bone-marrow derived dendritic cells (BMDC) for 12 hours (final concentration 5 nM DN**1**, therefore ~90 nM fluorophores). The Cy5 fluorescence intensity within the cells was measured using confocal microscopy at 12 hours post-washing.

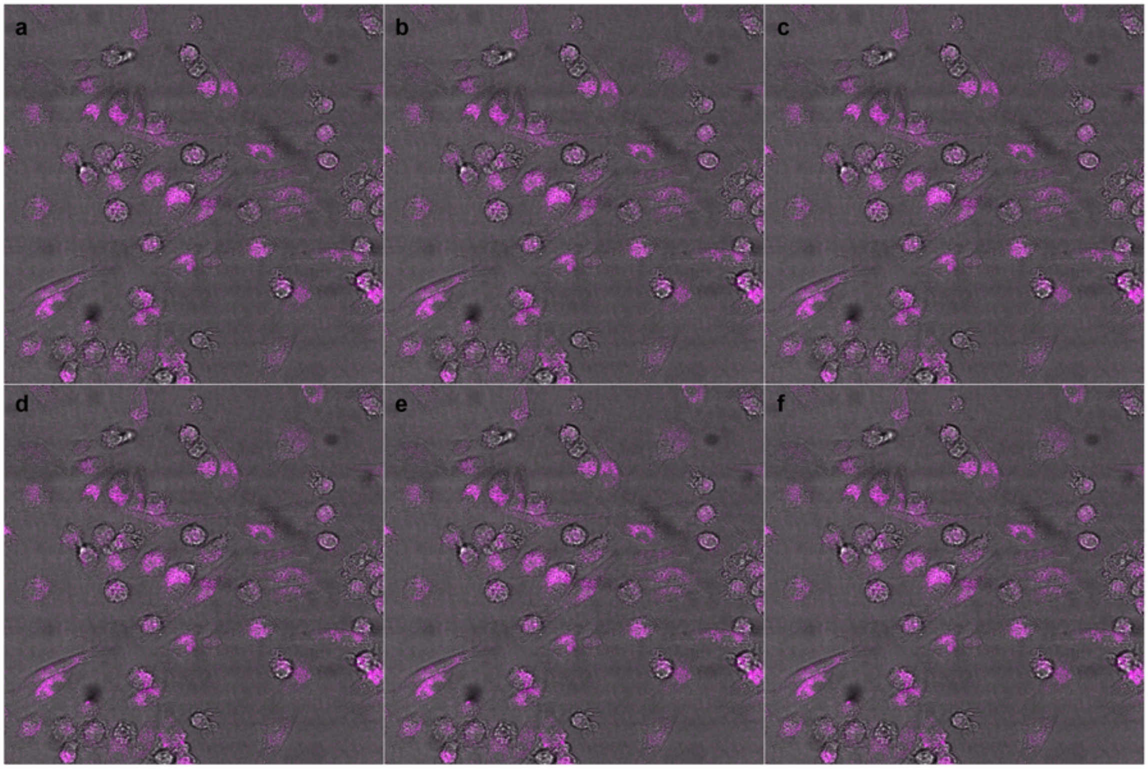

Supplementary Figure 29| K_{10}-PEG_{5K} coated structures are inside the cells and not stuck on the surface. K_{10}-PEG_{5K} coated Cy5 labelled DN**1** (5 nM, pink) was incubated with Bone-marrow dendritic cells (BMDC) for 12 hours, after which the medium was changed to remove DNs that were not taken up. The Cy5 fluorescence intensity within the cells was measured using confocal microscopy at 12 hours post-washing. Images (a-f) where **a** is at the top of the stack were collected at 0.2 μm intervals.

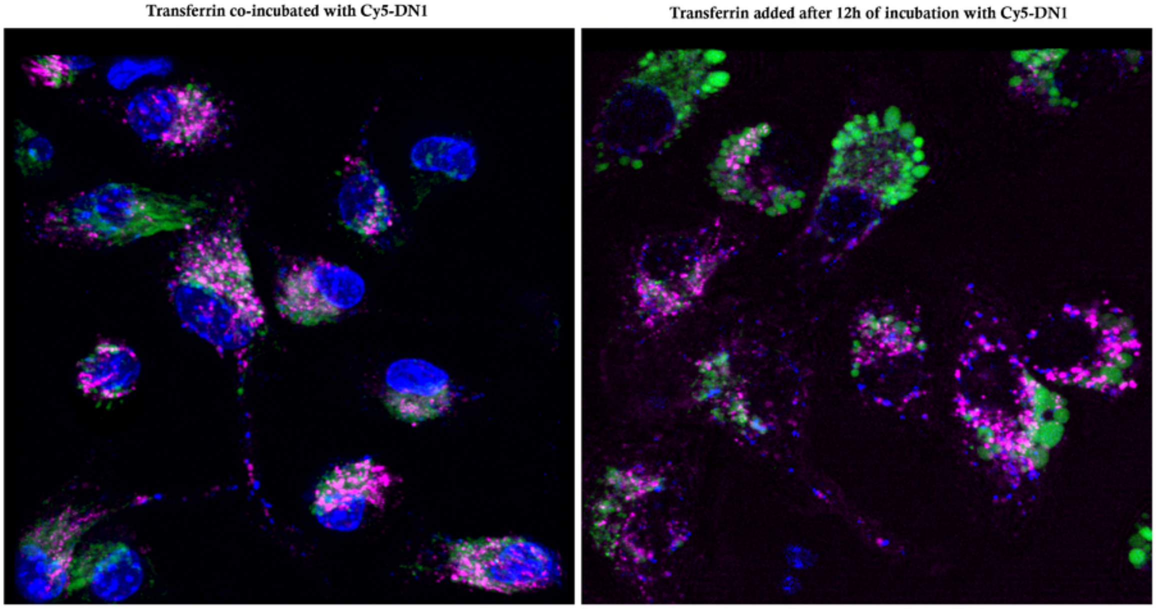

Supplementary Figure 30| K_{10}-PEG_{5K} coated Cy5 labelled DN1 are mostly localized in vesicular compartments such as endosomes and endo-lysosomes. Left: DN**1** (5 nM) and fluorescein-transferrin (green) was incubated with BMDC for 12 hours, after which nucleus-stain Hoechst (blue) was added and the sample was incubated for 1 hour prior to confocal imaging. **Right:** DN**1** (5 nM) was incubated with BMDC for 12 hours, after which fluorescein-transferrin (green) and nucleus-stain Hoechst (blue) was added and the sample was incubated for 1 hour prior to confocal imaging. We observe that K_{10}-PEG_{5K} coated Cy5 labelled DN**1** are mostly localized in vesicular compartments similar to fluorescein-transferrin.

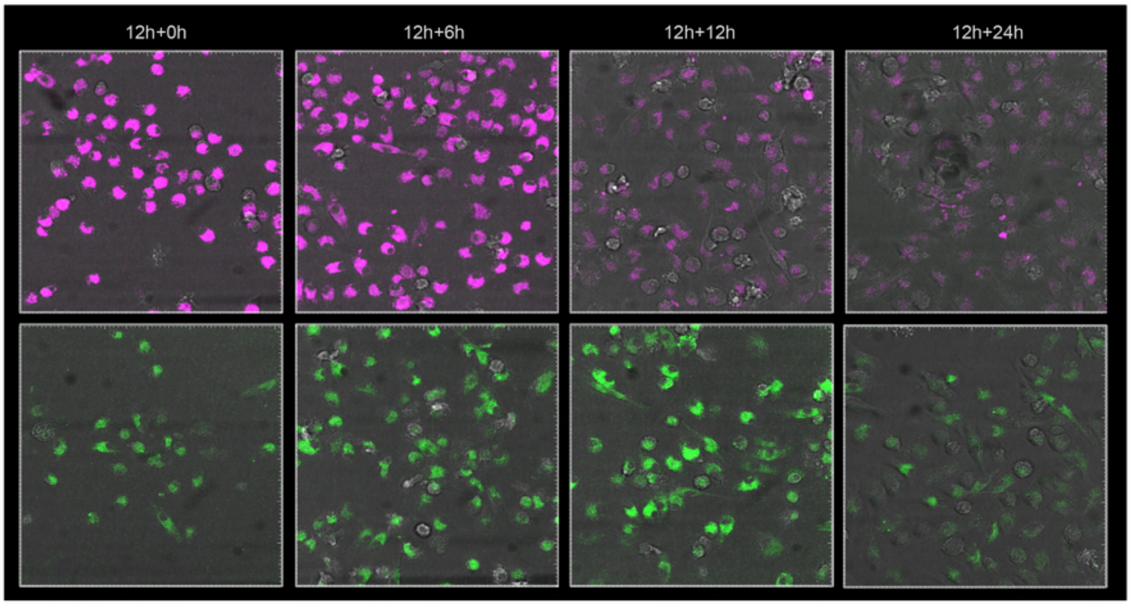

Time	Intensity	Std Dev
0 h	1.00	0.27
6 h	0.88	0.50
12 h	0.24	0.09
24 h	0.13	0.04

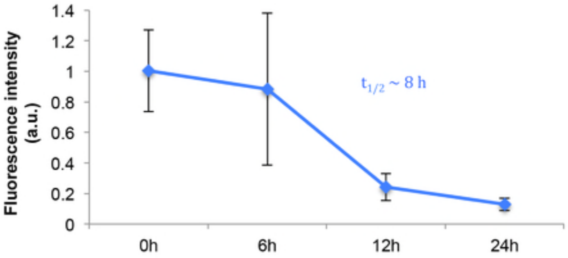

Supplementary Figure 31| Retention of Cy3 and Cy5 labelled DN1 within BMDC at 37 °C. DN**1** (5 nM) was incubated with BMDC for 12 hours, after which the medium was changed to remove DNs that were not taken up. The Cy3 and Cy5 fluorescence intensity within the cells was monitored *via* confocal at 0h, 6h, 12h and 24h. Top panel : Cy5 fluorophore (pink), bottom panel : Cy3 fluorophore (green). Half-life of DN**1** within BMDC is about 8 hours post-washing. Fluorescence intensity was calculated using ImageJ independently for both the channels and normalized to 0h time point. The error bars represent standard deviation.

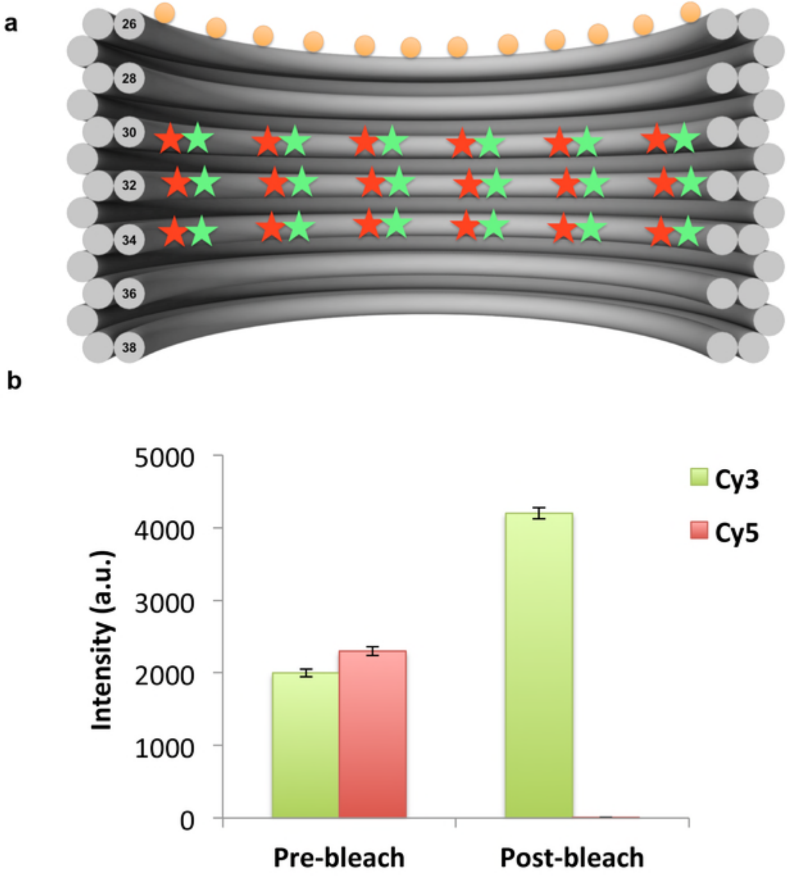

Supplementary Figure 32| Schematic representation of the cross section of DN**1** functionalized with 18 Cy3 (green stars) and 18 Cy5 (red stars) fluorophores and 12 biotins (yellow spheres). Biotin functionalization was used for the deposition of the DNs on a streptavidin coated glass surface. The samples were coated with K_{10}-PEG_{5K} at an N:P of 1:1. In order to simulate a cellular environment, the samples were incubated in BMDC culture medium. After ~99% photobleaching of Cy5 fluorophore, a 102.5% ± 2.4 enhancement in Cy3 fluorescence was observed. Error bars represent standard deviation.

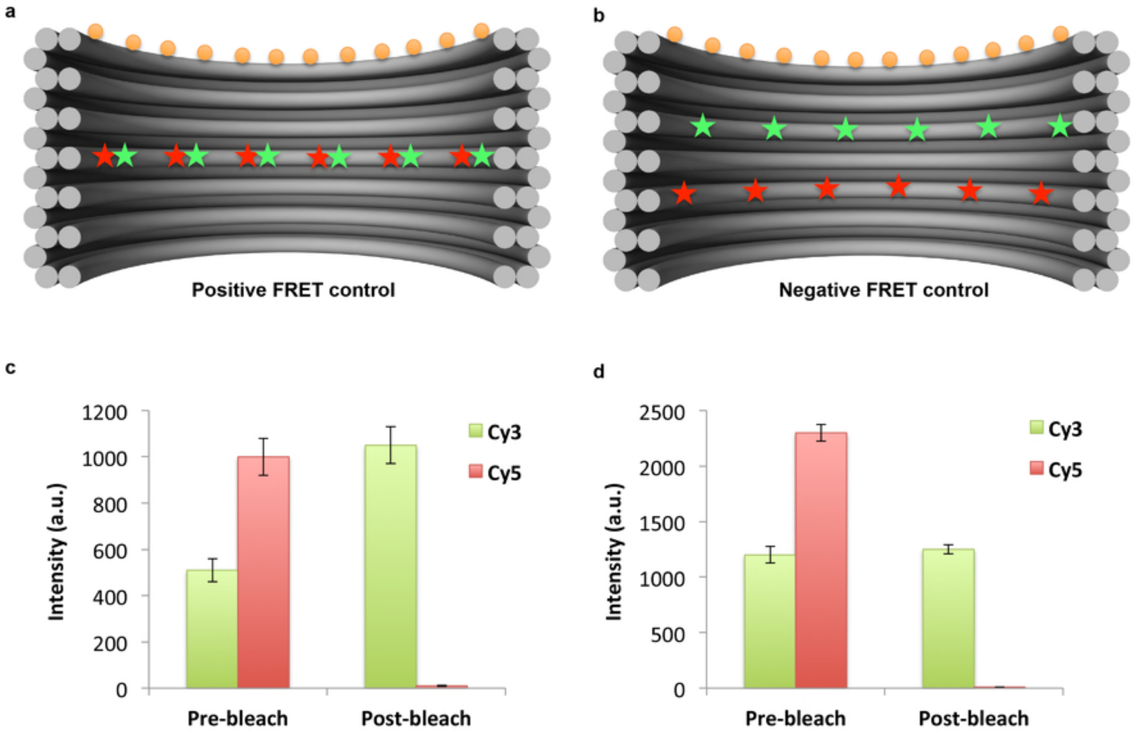

Supplementary Figure 33| Schematic representation of the cross section of DN**1** functionalized with 6 Cy3 (green stars) and 6 Cy5 (red stars) fluorophores and 12 biotins (yellow spheres). Biotin functionalization was used for the deposition of the DNs on a streptavidin coated glass surface. a) **Positive FRET control:** The Cy3 and Cy5 fluorophores are within Förster distance and can exhibit FRET. b) **Negative FRET control:** The Cy3 and Cy5 fluorophores are beyond Förster distance and cannot exhibit FRET. The samples were coated with K_{10}-PEG_{5K} at an N:P of 1:1. In order to simulate a cellular environment, the samples were incubated in BMDC culture medium. Enhancement of Cy3 signal after ~99% photobleaching of Cy5 fluorophore is only observed for the positive FRET system. Error bars represent standard deviation.

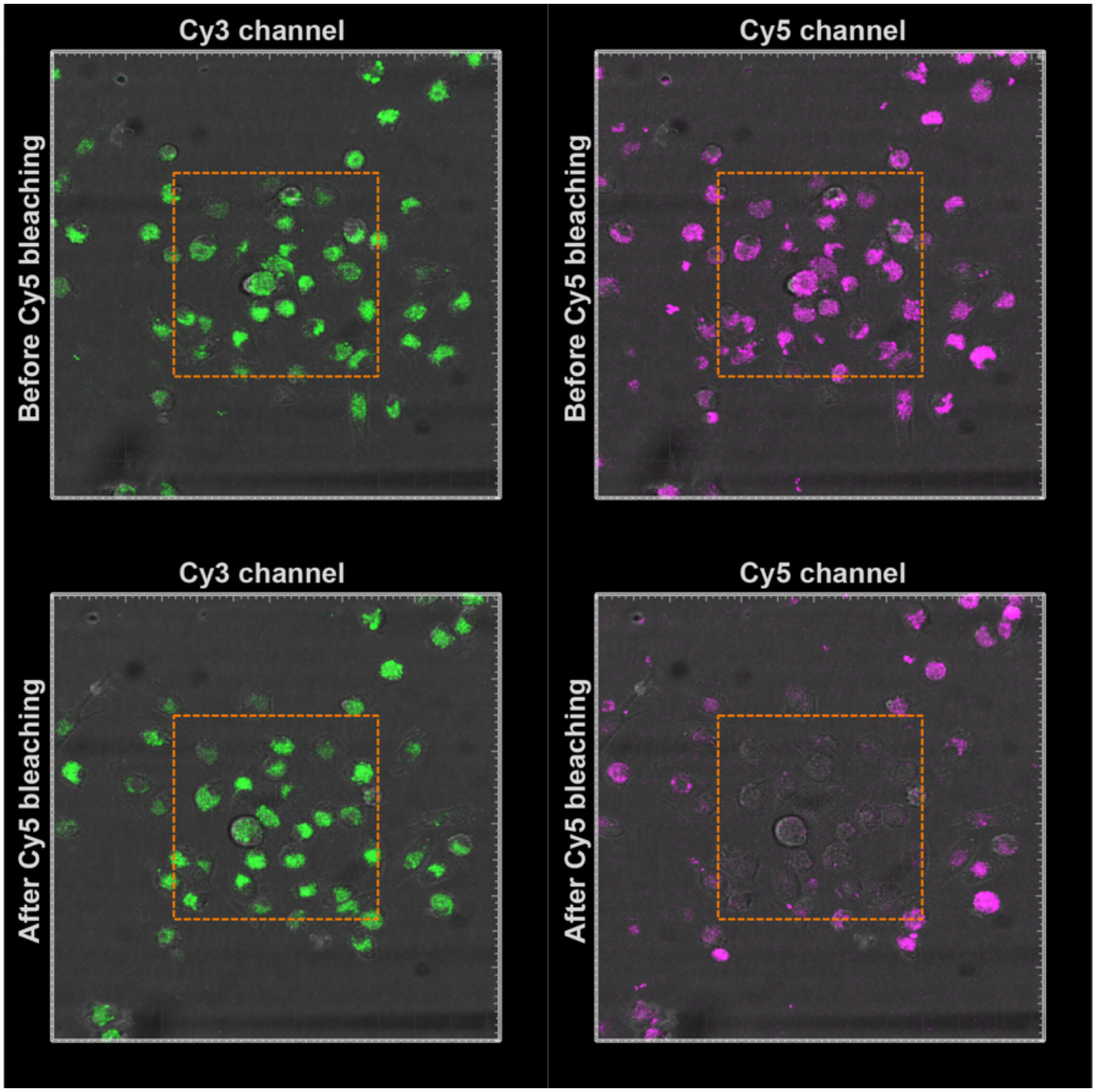

Supplementary Figure 34| Cy3 enhancement on Cy5 photobleaching within BMDC at 0h post-washing of cells. DN1 (5 nM) was incubated with BMDC for 12 hours, after which the medium was changed to remove DNs that were not taken up. The Cy3 and Cy5 fluorescence intensity within the cells post-washing before and after photobleaching of the Cy5 fluorophore at 0h.

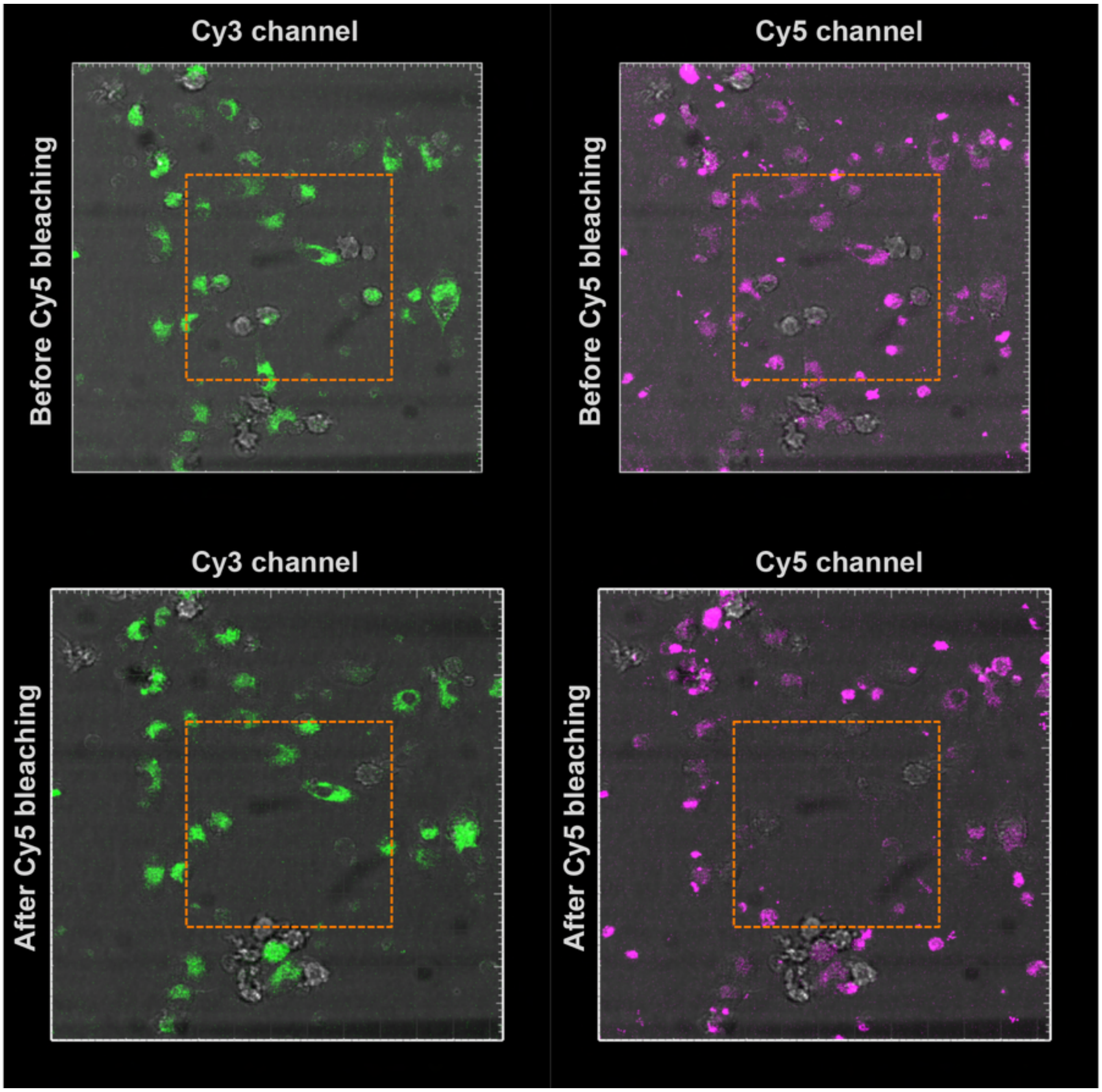

Supplementary Figure 35| Cy3 enhancement on Cy5 photobleaching within BMDC at 6h post-washing of cells. DN1 (5 nM) was incubated with BMDC for 12 hours, after which the medium was changed to remove DNs that were not taken up. The Cy3 and Cy5 fluorescence intensity within the cells post-washing before and after photobleaching of the Cy5 fluorophore at 6h.

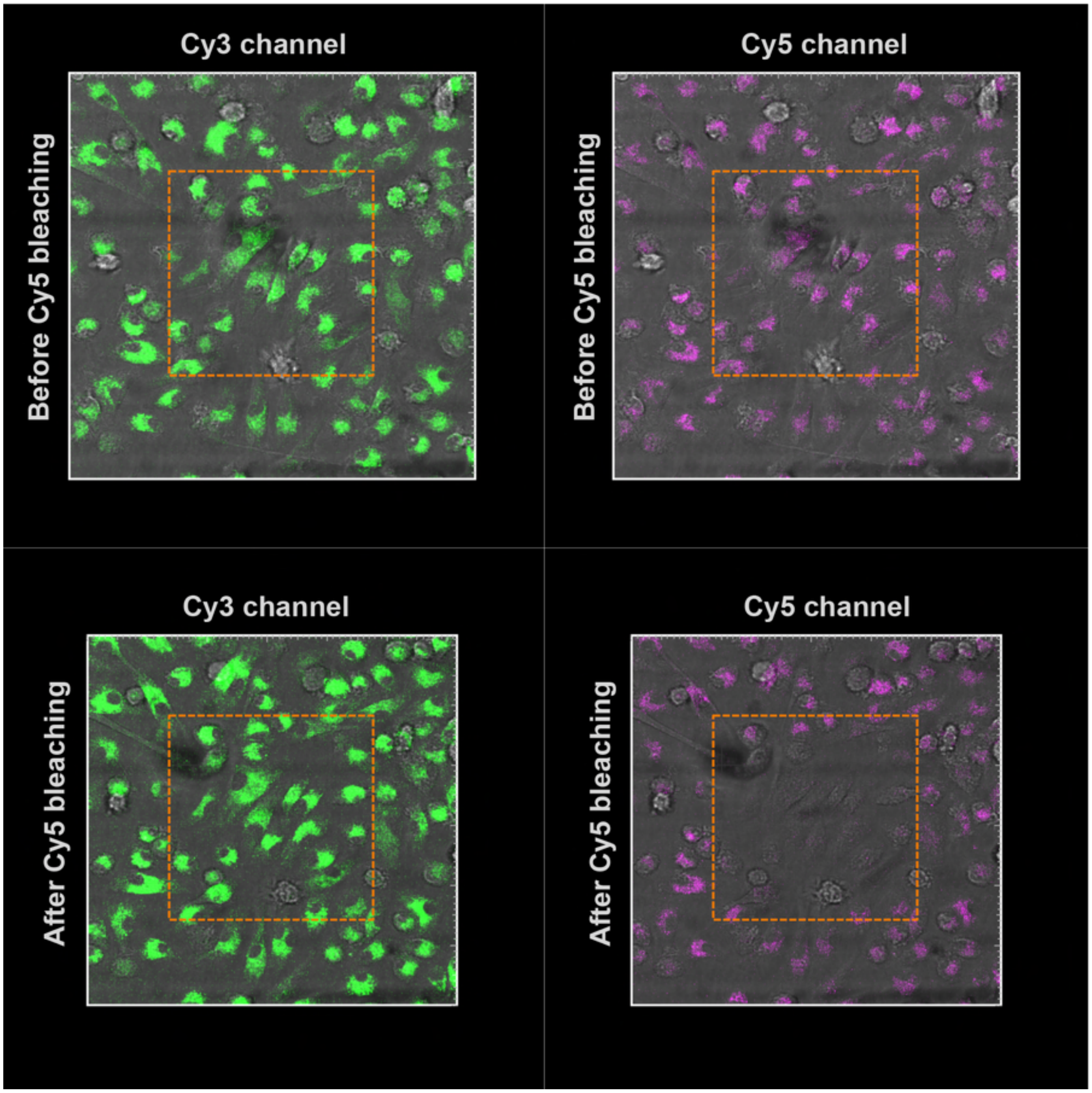

Supplementary Figure 36| Cy3 enhancement on Cy5 photobleaching within BMDC at 12h post-washing of cells. DN1 (5 nM) was incubated with BMDC for 12 hours, after which the medium was changed to remove DNs that were not taken up. The Cy3 and Cy5 fluorescence intensity within the cells post-washing before and after photobleaching of the Cy5 fluorophore at 12h.

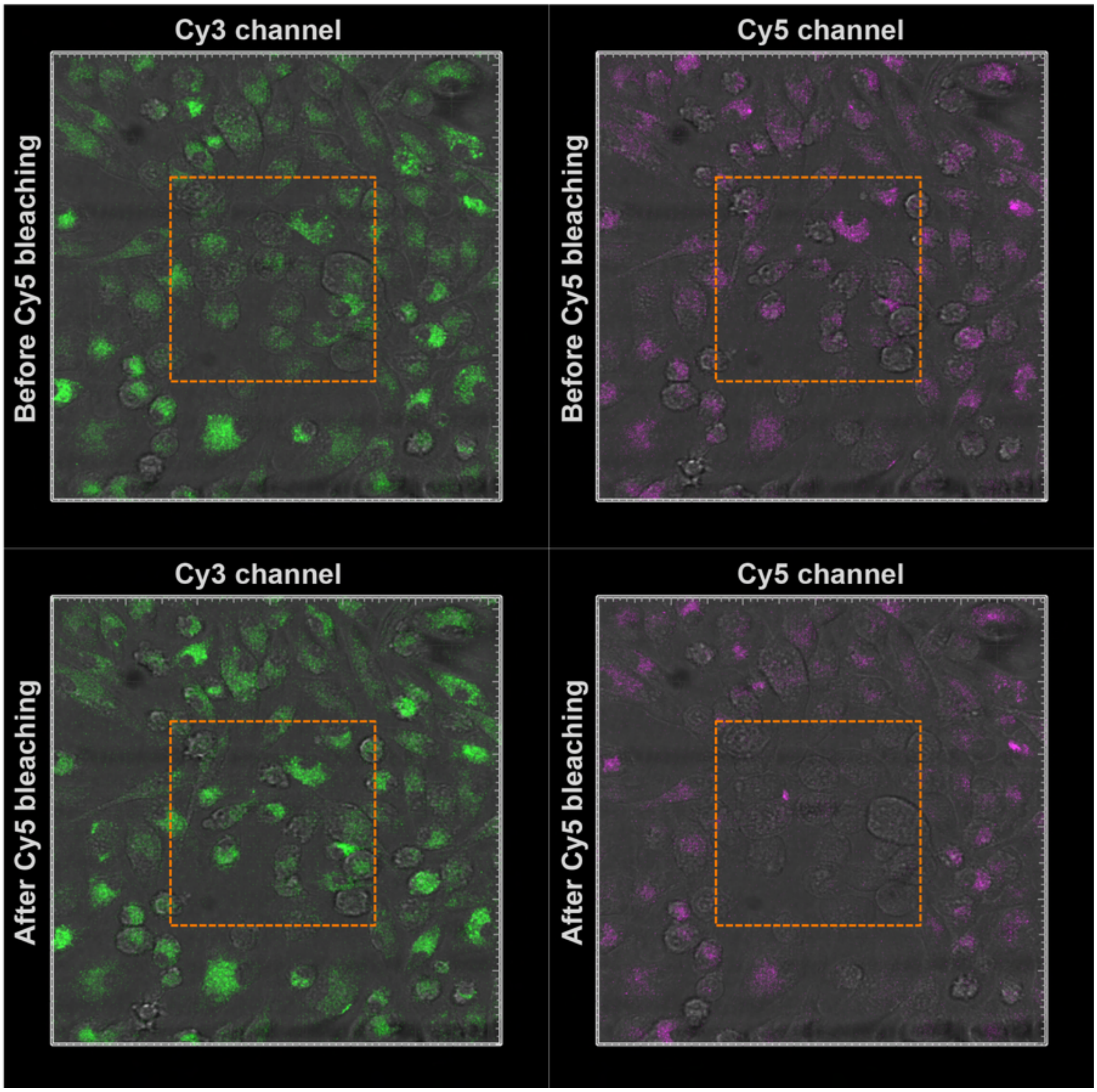

Supplementary Figure 37| Cy3 enhancement on Cy5 photobleaching within BMDC at 24h post-washing of cells. DN1 (5 nM) was incubated with BMDC for 12 hours, after which the medium was changed to remove DNs that were not taken up. The Cy3 and Cy5 fluorescence intensity within the cells post-washing before and after photobleaching of the Cy5 fluorophore at 24h.

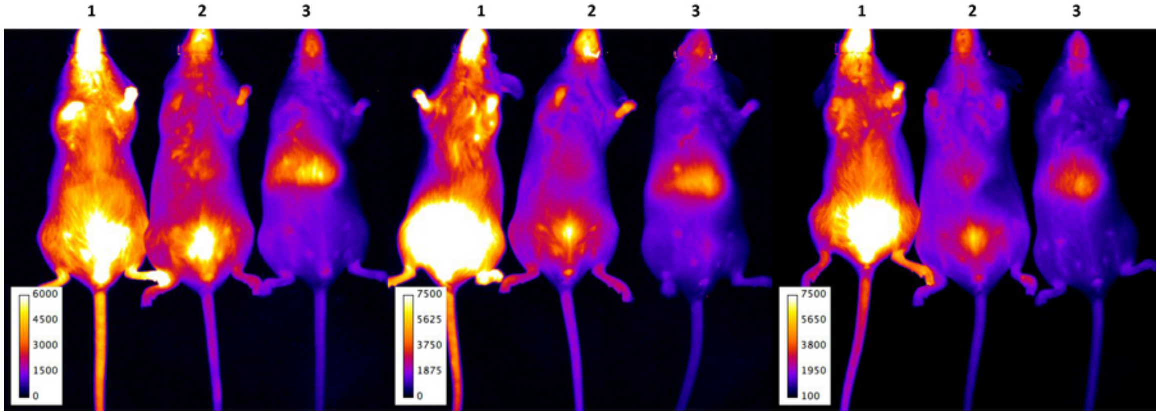

Supplementary Figure 38| Biodistribution profile of Alexa 750 labelled DN1 injected retro-orbitally in mice. Fluorescence images of mice injected with either (1) Alexa750 DNA oligo or (2) DN**1**$_{bare}$ or (3) DN**1**$_{coated}$ functionalized with 36 Alexa750 fluorophores at 2 minutes post injection. Alexa 750 fluorescence was monitored every 2 minutes for 1 hour using Perkin Elmer In Vivo Imaging System (IVIS).

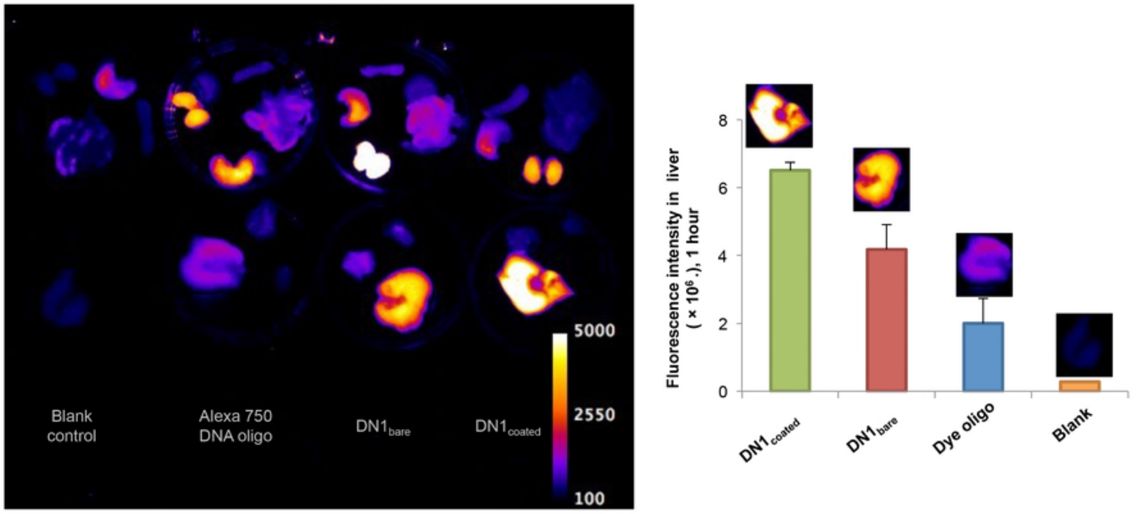

Supplementary Figure 39| Distribution of fluorescence in the mice organs. The mice were injected with either (1) Alexa750 DNA oligo or (2) DN**1**$_{bare}$ or (3) DN**1**$_{coated}$ functionalized with 36 Alexa750 fluorophores. The mice organs were harvested and analyzed 1h post injection. K_{10}-PEG_{5K} coated DN**1** showed the highest accumulation in the liver. Error bars represent standard deviation (n=3).

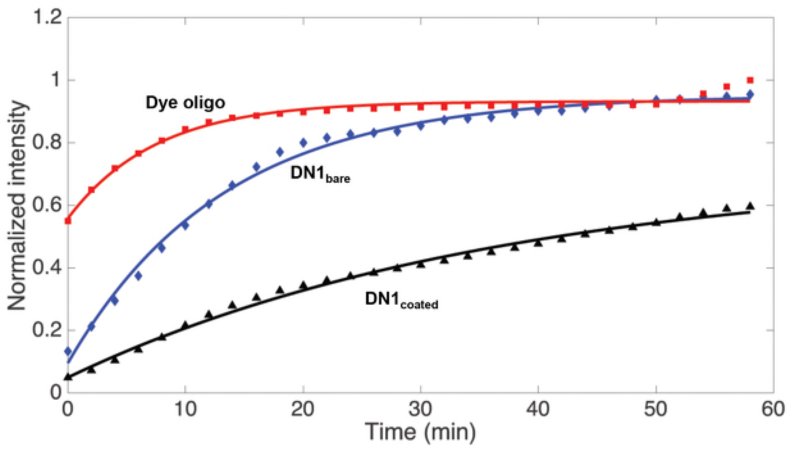

Sample	t1/2
Dye oligo	5.19
DN1 bare	9.08

Supplementary Figure 40| Fitting of the biodistribution data. The fluorescence intensity of the dye oligo, DN**1**$_{bare}$ and DN**1**$_{coated}$ was normalized to the to the maximum fluorescence intensity observed in the bladder for the dye oligo. The data of dye oligo and DN**1**$_{bare}$ was fitted to an exponential using MATLAB. For DN**1**$_{coated}$, the data fit is not very reliable since it did not reach 100% saturation in the bladder at 60 minutes; we simply take the 50% normalized intensity as the provisional renal clearance half-life of ~45 min in this case.

**Model Fit**	**Goodness of fit**
**Dye oligo**	sse: 0.0097
**result(x) = a*exp(-b*x)+c**	rsquare: 0.9630
Coefficients (with 95% confidence bounds):	dfe: 27
a = -0.3743 (-0.4061, -0.3424)	adhrsquare: 0.9603
b = 0.1335 (0.1114, 0.1557)	rmse: 0.0190
c = 0.9321 (0.9221, 0.9422)	
**Bare DN1**	sse: 0.0096
**result(x) = a*exp(-b*x)+c**	rsquare: 0.9940
Coefficients (with 95% confidence bounds):	dfe: 27
a = -0.8563 (-0.8832, -0.8295)	adhrsquare: 0.9936
b = 0.07594 (0.0702, 0.0817)	rmse: 0.0189
c = 0.952 (0.9367, 0.9672)	

Supplementary Figure 41| caDNAno for DN1

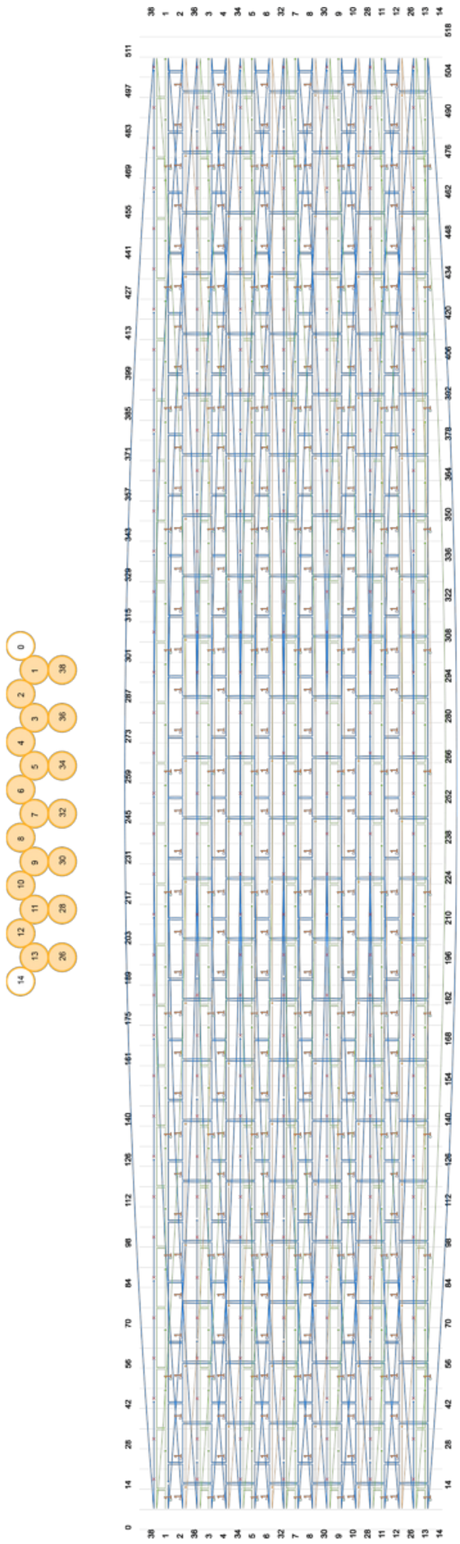

Supplementary Figure 42| caDNAno for DN2

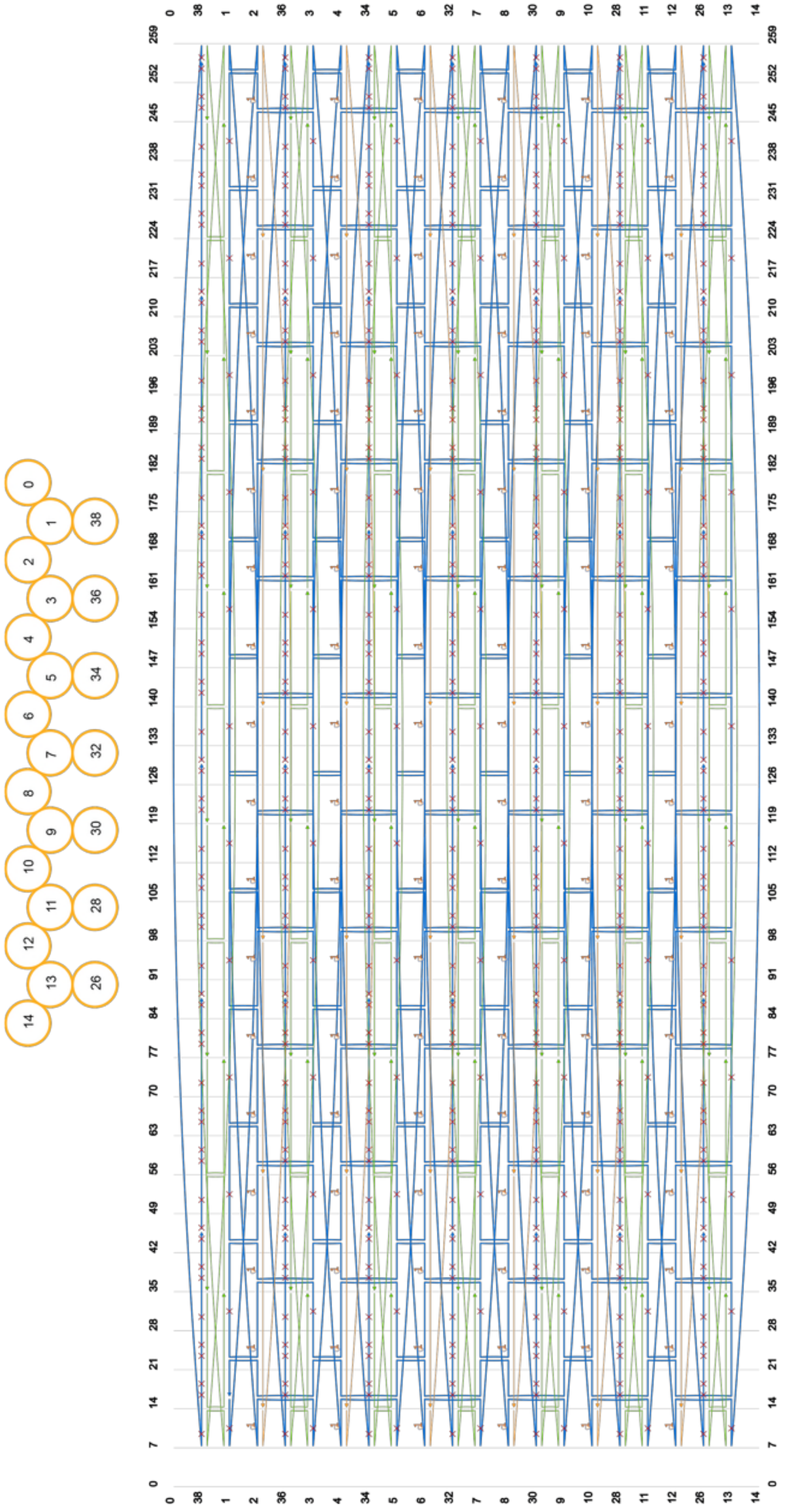

Supplementary Figure 43| caDNAno for DN3

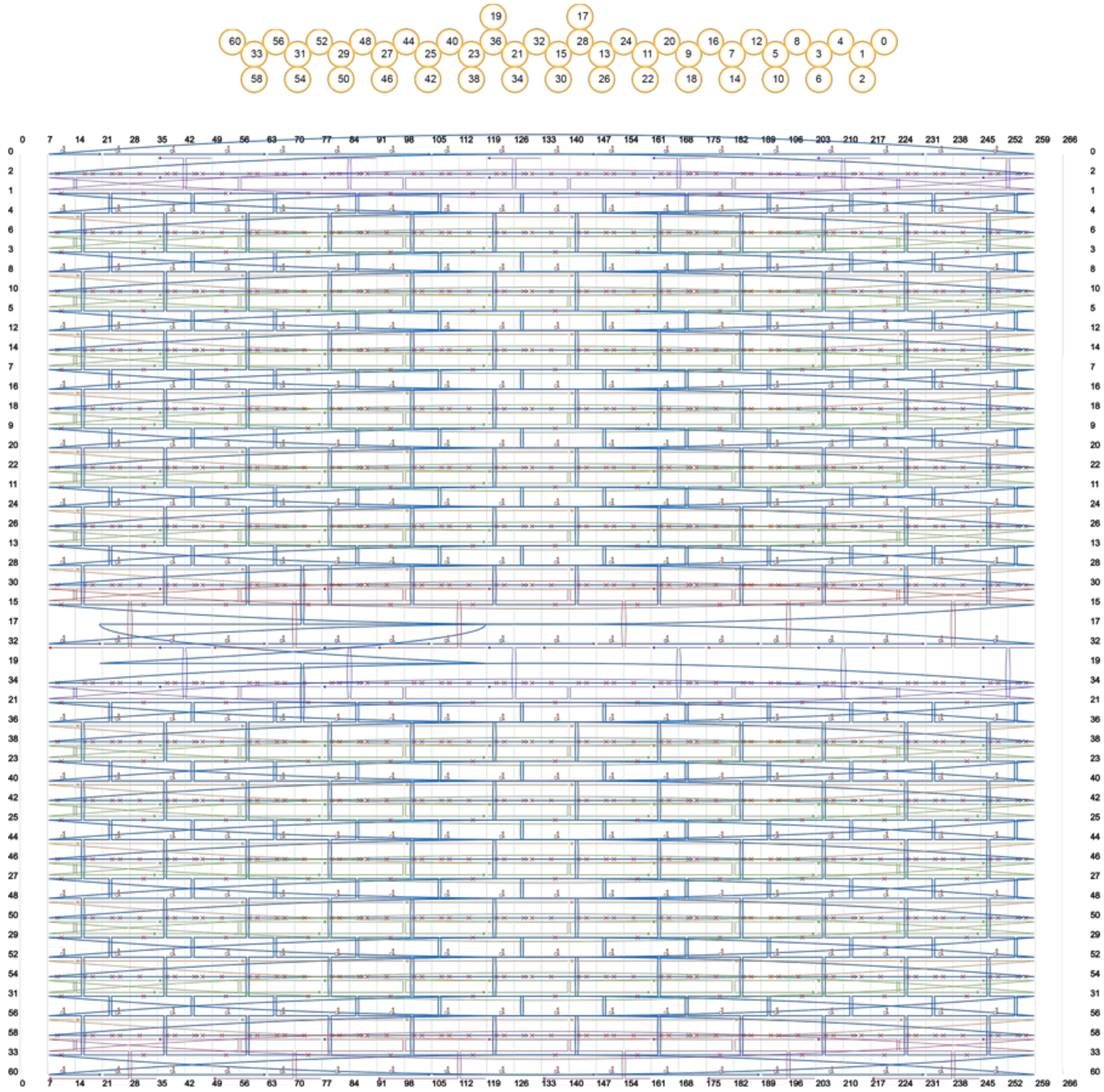

Supplementary Figure 44| caDNAno for DN4

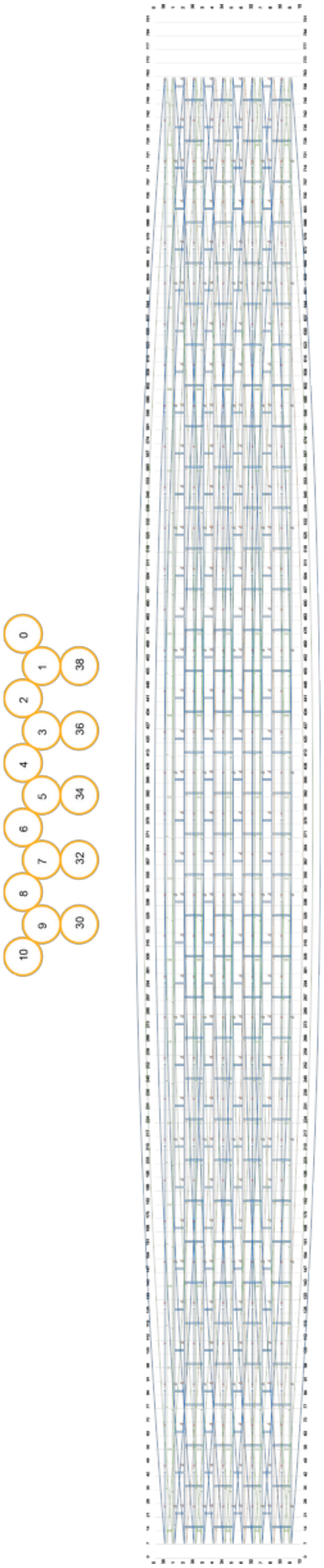

Supplementary Figure 45| caDNAno for DN5

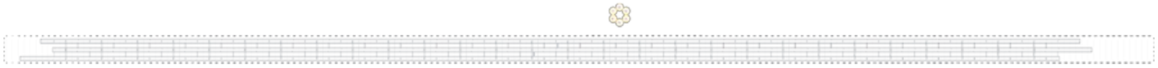

Supplementary Figure 46| caDNAno for DN6

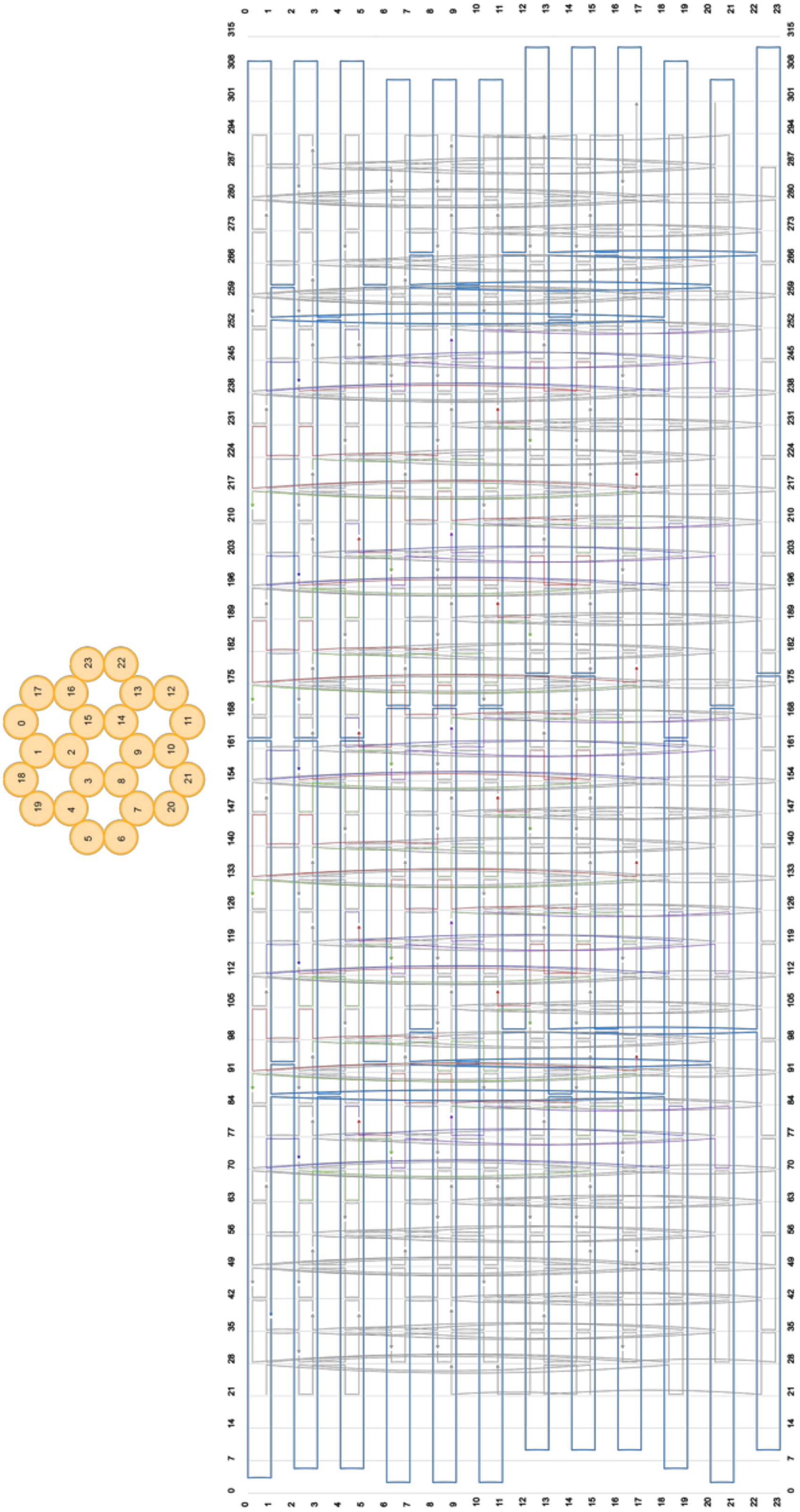

Supplementary Figure 47| caDNAno for DN7

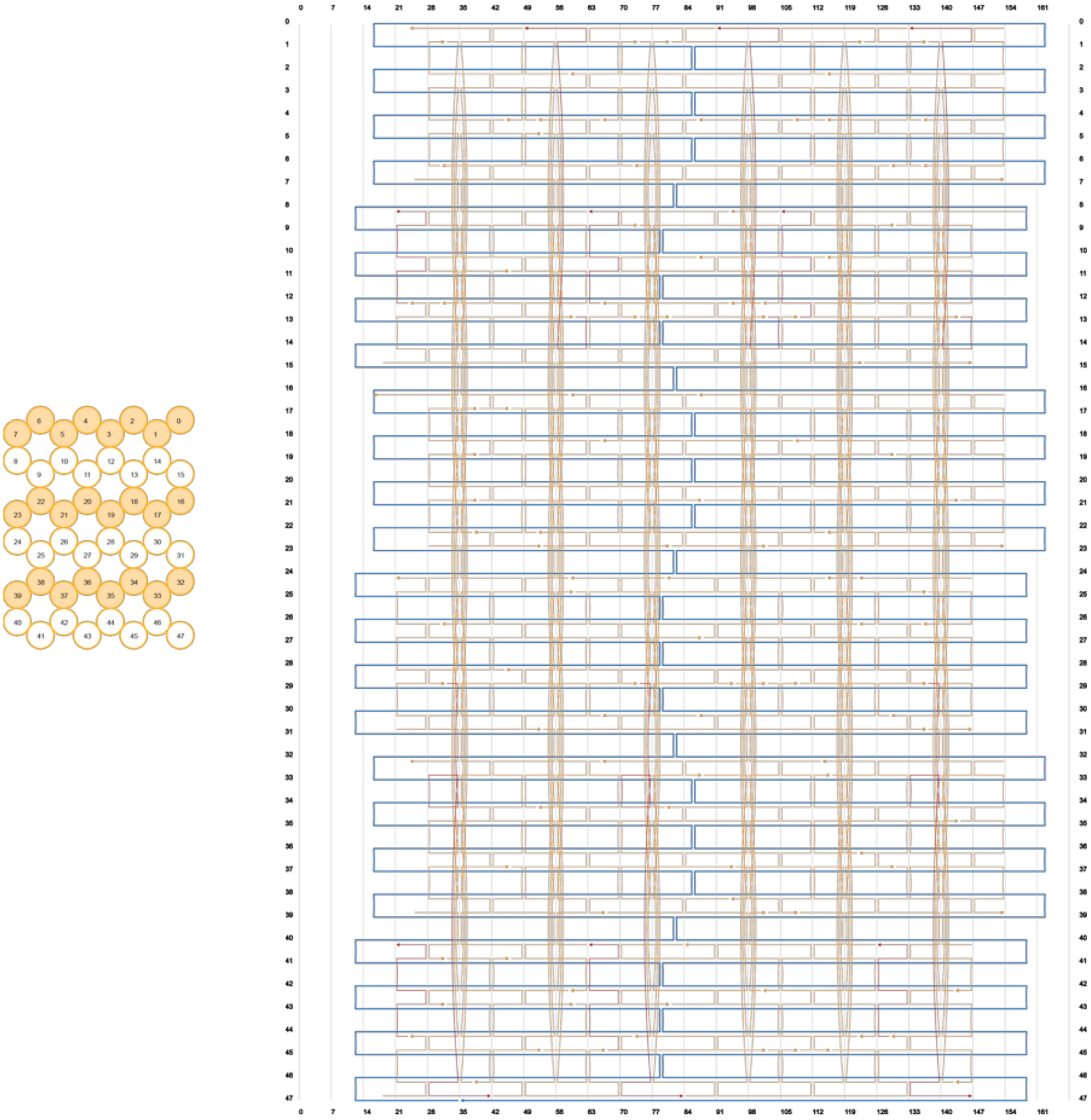

Supplementary Figure 48| caDNAno for DN8

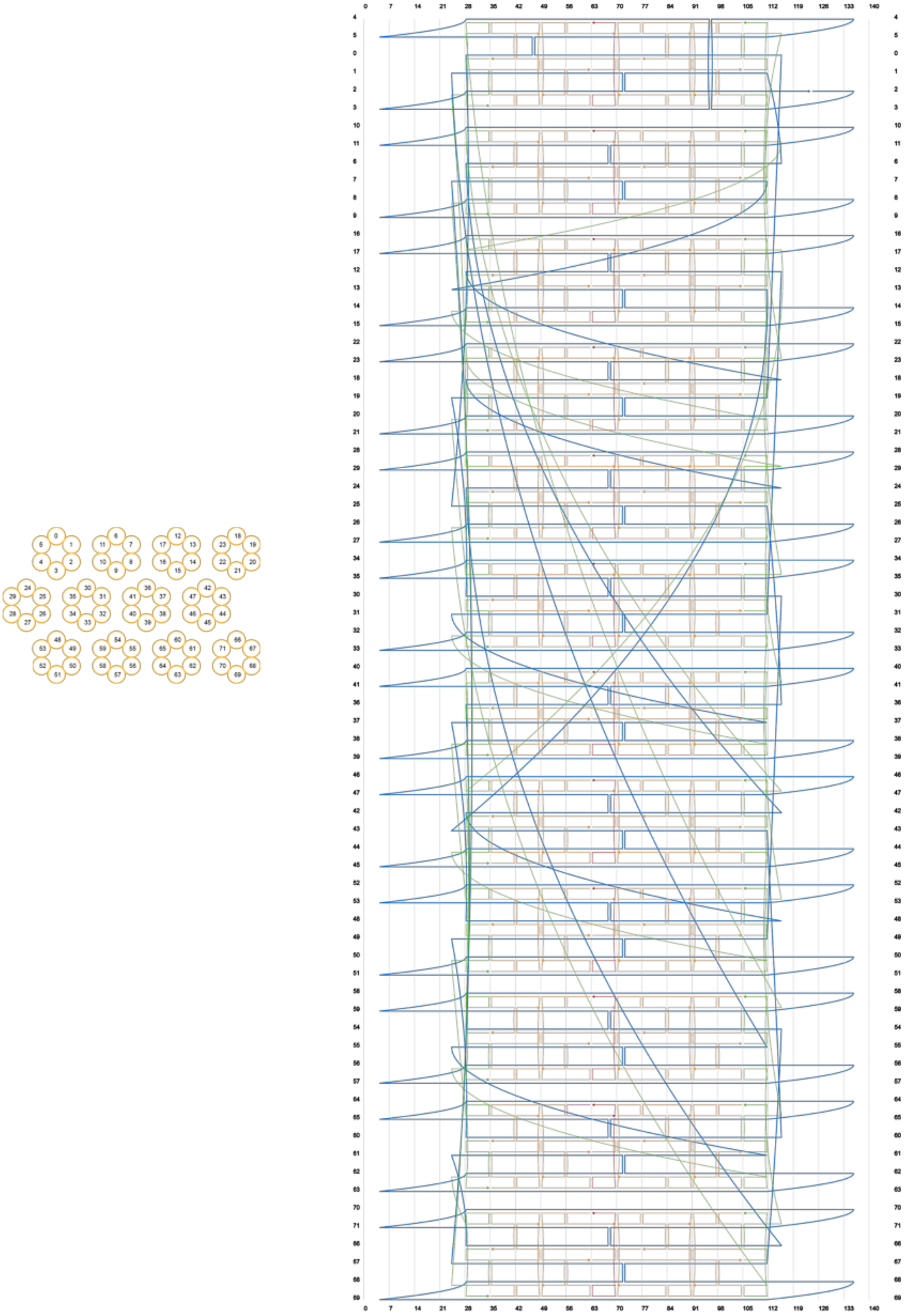

SUPPLEMENTARY TABLES

	**Na^+**	**K^+**	**Mg^{2+}**	**Ca^{2+}**
DN folding Buffer (0.5x TE + Mg^{2+})	-	-	6 - 20 mM	-
1x PBS	146 mM	6.9 mM	-	-
RPMI-1640	130 mM	10 mM	0.4 mM	0.4 mM
DMEM	154 mM	10 mM	0.8 mM	1.8 mM
Serum	~135 mM	~3.5 mM	~0.6 mM	~1 mM
Cytosol	~10 mM	~140 mM	~1.0 mM	~100 nM

Supplementary Table 1| Concentrations of monovalent and divalent cations in the folding buffer of DNA nanostructures (DN) and various physiological buffers. The folding buffer of DNs typically requires 6 − 20 mM Mg^{2+}, however, physiological fluids typically contain low-levels of divalent cations. The cation concentration values for RPMI-1640 and DMEM were obtained from the product specifications.

SUPPLEMENTARY NOTES

Supplementary Note 1

In our cell experiments (HeLa, HUVE and BMDC) DNs were not found to be toxic at a maximum tested concentration of 10 nM.

We measured cell viability using the Thermofisher live/dead cell imaging kit. The live cell component produces an intense, uniform green fluorescence in live cells (ex/em 488 nm/515 nm). The dead cell component produces a predominantly nuclear red fluorescence (ex/em 570nm/602 nm) in cells with compromised cell membranes, a strong indicator of cell death and cytotoxicity (Supplementary Figure 41, 42).

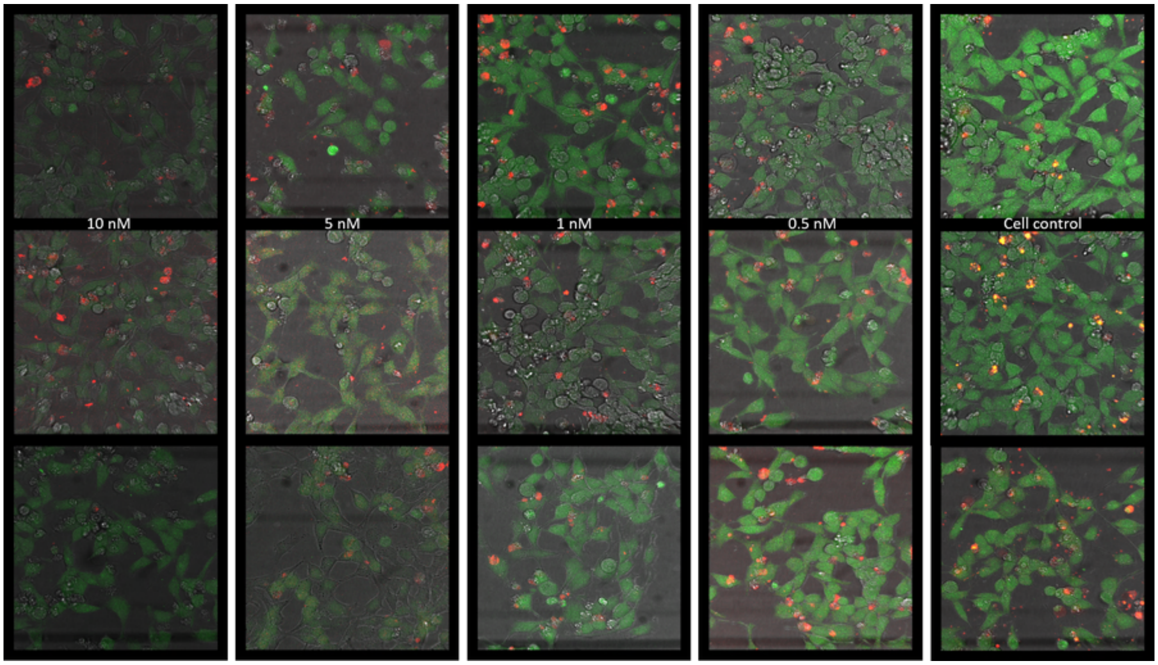

Supplementary Figure 49| Live/dead imaging of HeLa cells. Cells were incubated with 0.5 nM – 10 nM DN in cell medium for 12 hours after which the live/dead dual probe was added. Cells were imaged using confocal microscopy at 488nm and 570nm.

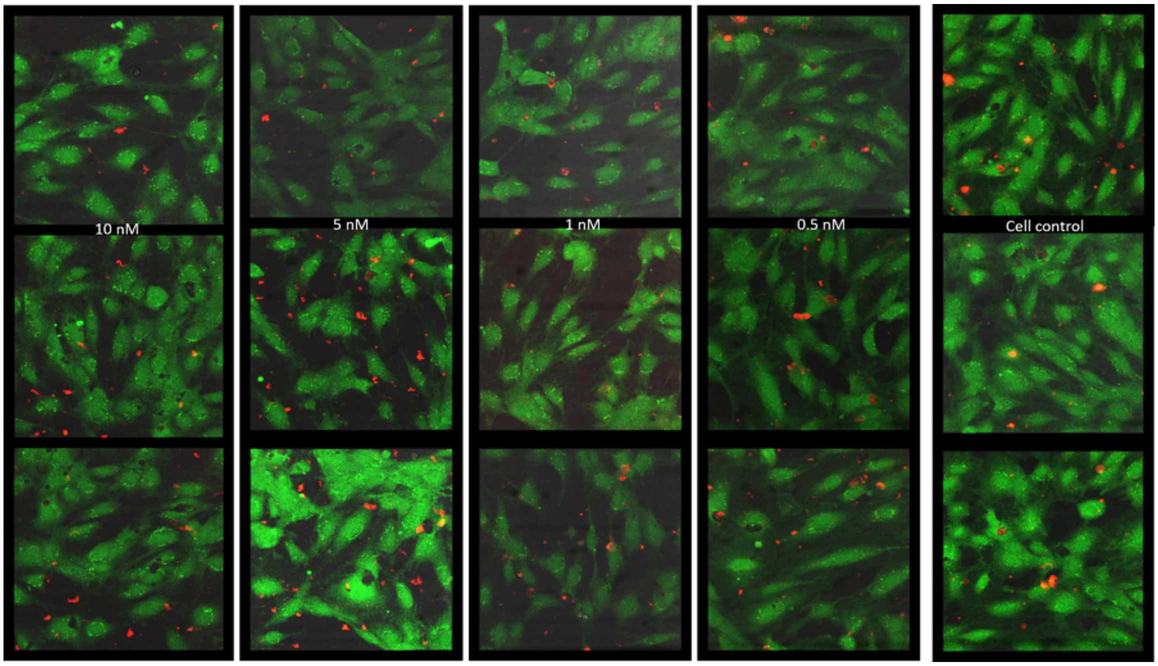

Supplementary Figure 50| Live/dead imaging of HUVE cells. Cells were incubated with 0.5 nM – 10 nM DN in cell medium for 12 hours after which the live/dead dual probe was added. Cells were imaged using confocal microscopy at 488nm and 570nm.

Supplementary Note 2

To validate that our FRET system could be used to monitor structural integrity, we measured the magnitude of Cy3 fluorescence enhancement induced by disruption of the arrangement between Cy3 and Cy5 dyes. For this initial experiment, the disruption was induced via treatment with 100 mM EDTA (Ethylenediaminetetraacetic acid) for 30 min at 37ºC, and fluorescence was monitored for particles in bulk solution. This treatment resulted in Cy3 fluorescence enhancement of ~700%. Conversely, for a negative control labelled with only Cy3, only 40% fluorescence enhancement was observed (Supplementary Fig. 33). This result established reference behaviour for intact structures due to EDTA treatment of 700% Cy3 fluorescence. For example, a structure in which half the Cy3 dyes have lost their Cy5 partner, e.g. due to denaturation or degradation, would be expected to exhibit only 350% Cy3 fluorescence enhancement following EDTA treatment.

Next we examined a time course of DNAse I digestion, monitored via agarose-gel analysis, TEM analysis, and Cy3 fluorescence analysis, to observe how degradation of structure accumulated up to that time point correlated with Cy3 fluorescence enhancement induced by EDTA added after that time point (i.e. any loss of FRET induced by prior DNAse I degradation reduces the amount Cy3 fluorescence enhancement due to subsequent EDTA denaturation). We incubated DN**1**$_{coated}$ with RPMI-1640 containing 100U/mL DNase I for different time points at 37ºC. In order to monitor the recovery of Cy3 fluorescence upon DNase I degradation, we first deactivated the DNase I using EGTA and β-mercaptoethanol, stripped the K_{10}-PEG_{5K} shell using chondroitin sulfate and then treated our samples with 100 mM EDTA for 30 min at 37ºC to induce denaturation. We note that at the $t_{1/2}$ for loss of fluorescence enhancement (6 hours in this system), structures were still intact as observed by AGE and TEM (Supplementary Fig 37). Unravelling of particles only was observed after ~70% of the fluorescence enhancement had been lost. This is consistent with a model in which DNA nanostructures can sustain many local lesions before global collapse occurs.

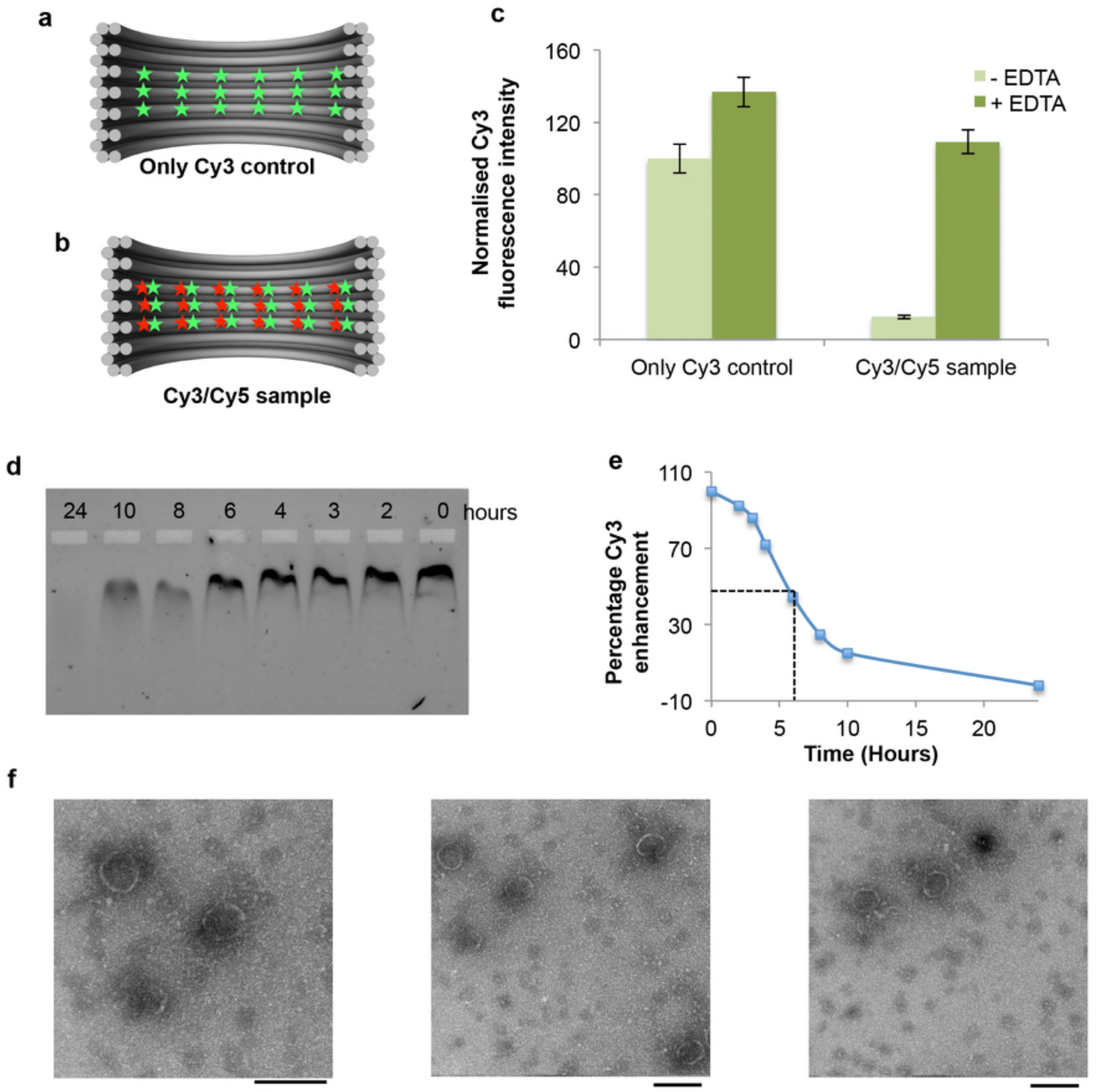

Supplementary Figure 51| DNs are intact at 50% fluorescence enhancement. a) DN**1** labelled with 18 Cy3 fluorophores. b) DN**1** labelled with 18 Cy3 and 18 Cy5 fluorophores. c) Cy3 fluorescence intensity before and after addition of EDTA (error bars represent standard deviation, n=2) d) DN**1** coated with K_{10}-PEG_{5K} was incubated with RPMI-1640 containing 100U/mL DNase I for different time points at 37ºC and analysed via AGE. In order to monitor the recovery of Cy3 fluorescence upon DNase I degradation, DNase I was first deactivated using 5 mM EGTA and 10% β-mercaptoethanol, the K_{10}-PEG_{5K} shell was stripped using 100x chondroitin sulfate and then samples were treated with 100 mM EDTA for 30 min at 37ºC to induce denaturation. e) Percentage Cy3 enhancement upon addition of EDTA to the above samples. f) Negative stain TEM images of DN**1** at 6h timepoint. At $t_{1/2}$ for loss of fluorescence enhancement (6 hours), structures were still intact as observed by TEM (crude samples were used for TEM imaging). This shows that DNA nanostructures can sustain many local cuts before global collapse occurs. Scale bars are 100 nm.

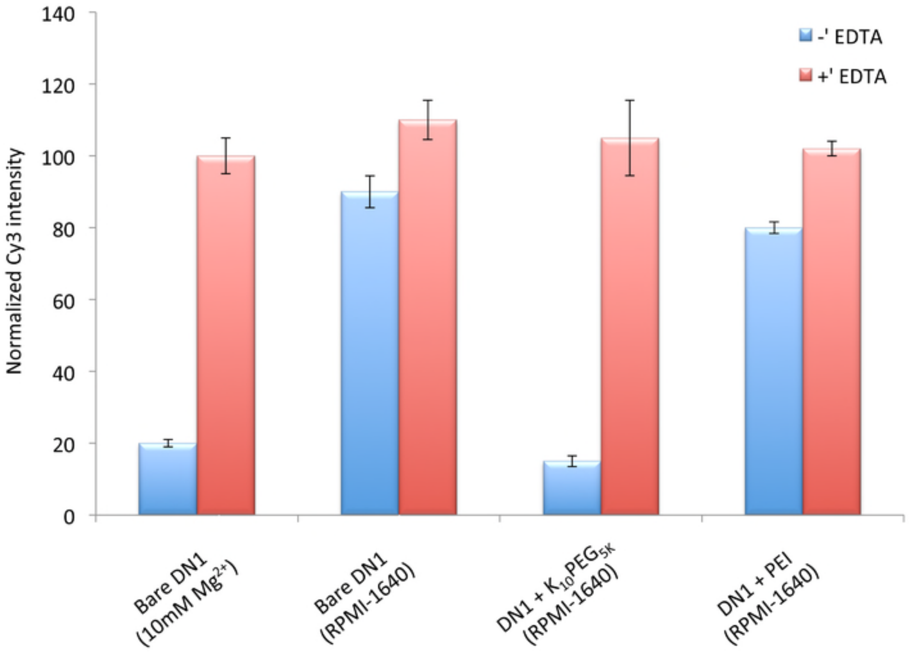

Supplementary Figure 52| Enhancement in Cy3 intensity upon EDTA denaturation is only observed when the DNA nanostructure is intact. We did this experiment to confirm that enhancement in Cy3 signal within cells is only observed when the structures are intact and not when they are compacted in the endosomal compartments. Cy3 intensity before and after addition of EDTA is plotted for four samples. Only structurally intact nanostructures show enhancement in Cy3 signal upon denaturation whereas nanostructures compacted with polyethyleneimine do not. Error bars represent standard deviation (n=2).

TEM images shows that PEI causes aggregation and irreversibly condenses the DNA nanostructure. The aggregation caused by PEI was not reversed even on addition of chondroitin sulfate.

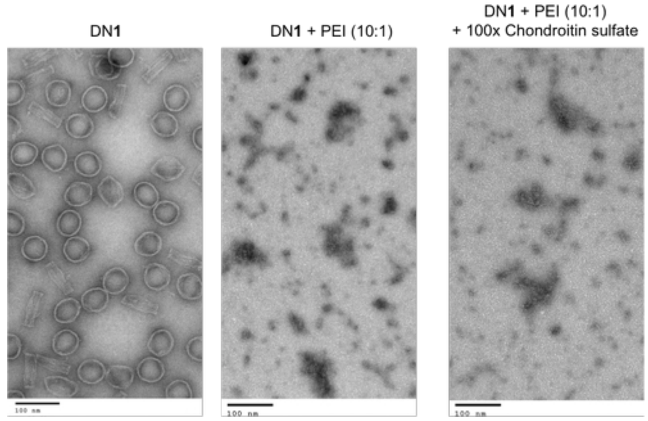

Supplementary Figure 53| TEM image of DN1 after addition and removal of PEI. (Left) bare DN1; (center) DN1 coated with 10:1 PEI and (right) DN1 coated with PEI and incubated with 100x chondroitin sulfate. Scale bars are 100 nm.
